# Maturation of Auditory Cortex Neural Activity in Children and Implications for Auditory Clinical Markers in Diagnosis

**DOI:** 10.3389/fpsyt.2020.584557

**Published:** 2020-11-19

**Authors:** J. Christopher Edgar, Lisa Blaskey, Heather L. Green, Kimberly Konka, Guannan Shen, Marissa A. Dipiero, Jeffrey I. Berman, Luke Bloy, Song Liu, Emma McBride, Matt Ku, Emily S. Kuschner, Megan Airey, Mina Kim, Rose E. Franzen, Gregory A. Miller, Timothy P. L. Roberts

**Affiliations:** ^1^Department of Radiology, Lurie Family Foundations Magnetoencephalography Imaging Center, The Children's Hospital of Philadelphia, Philadelphia, PA, United States; ^2^Department of Radiology, Perelman School of Medicine, University of Pennsylvania, Philadelphia, PA, United States; ^3^Department of Pediatrics, Center for Autism Research, The Children's Hospital of Philadelphia, Philadelphia, PA, United States; ^4^Department of Psychiatry, Perelman School of Medicine, University of Pennsylvania, Philadelphia, PA, United States; ^5^Department of Psychology, University of California, Los Angeles, Los Angeles, CA, United States; ^6^Department of Psychiatry and Biobehavioral Sciences, University of California, Los Angeles, Los Angeles, CA, United States

**Keywords:** auditory, pediatric, maturation, M50, M100, auditory, magnetoencephalography (MEG)

## Abstract

Functional brain markers that can inform research on brain abnormalities, and especially those ready to facilitate clinical work on such abnormalities, will need to show not only considerable sensitivity and specificity but enough consistency with respect to developmental course that their validity in individual cases can be trusted. A challenge to establishing such markers may be individual differences in developmental course. The present study examined auditory cortex activity in children at an age when developmental changes to the auditory cortex 50 ms (M50) and 100 ms (M100) components are prominent to better understand the use of auditory markers in pediatric clinical research. MEG auditory encoding measures (auditory evoked fields in response to pure tone stimuli) were obtained from 15 typically developing children 6–8 years old, with measures repeated 18 and 36 months after the initial exam. MEG analyses were conducted in source space (i.e., brain location), with M50 and M100 sources identified in left and right primary/secondary auditory cortex (Heschl's gyrus). A left and right M50 response was observed at all times (Time 1, Time 2, Time 3), with M50 latency (collapsing across hemisphere) at Time 3 (77 ms) 10 ms earlier than Time 1 (87 ms; *p* < 0.001) and with M50 responses on average (collapsing across time) 5 ms earlier in the right (80 ms) than left hemisphere (85 ms; *p* < 0.05). In the majority of children, however, M50 latency changes were not constant across the three-year period; for example, whereas in some children a ~10 ms latency reduction was observed from Time 1 to Time 2, in other children a ~10 ms latency reduction was observed from Time 2 to Time 3. M100 responses were defined by a significant “peak” of detected power with magnetic field topography opposite M50 and occurring 50–100 ms later than the M50. Although M100s were observed in a few children at Time 1 and Time 2 (and more often in the right than left hemisphere), M100s were not observed in the majority of children except in the right hemisphere at Time 3. In sum, longitudinal findings showed large between- and within-subject variability in rate of change as well as time to reach neural developmental milestones (e.g., presence of a detectable M100 response). Findings also demonstrated the need to examine whole-brain activity, given hemisphere differences in the rate of auditory cortex maturation. Pediatric research will need to take such normal variability into account when seeking clinical auditory markers.

## Introduction

Researchers conducting EEG and MEG clinical studies have long sought to identify auditory brain measures that predict patient group status. Especially prominent are studies seeking to identify auditory encoding and gating deficits in adults with schizophrenia (1–13), and studies seeking to identify auditory encoding and auditory discrimination deficits in children and adults with autism spectrum disorder (14–21). As detailed in Edgar (22), control and case group differences in the rate of brain maturation may constrain the use of brain markers. As an example, given normal maturation of a brain measure in controls and abnormally slow maturation in cases, the use of the measure to differentiate groups may be of use only for a certain age range.

A different view of auditory neural measures reveals a shifting landscape of auditory measures across the lifespan, with some evoked components such as the 50 ms auditory response thought to be present throughout life, some responses such as the 100 ms response appearing only by early adolescence (see details in Discussion), and some responses such as the 40 Hz steady-state response not reliably observed until late adolescence [e.g., see (23–26)]. As such, although several different auditory components are of interest, as they allow assessment of different aspects of auditory cortex neural function (see Discussion), selection of auditory brain markers in clinical pediatric studies is often—or should be—constrained by the age of the sample. And for each component of interest, information regarding the rate of maturation in typically developing populations is lacking, information that is needed in order to identify abnormal maturation in patient populations.

The present study sought to begin to understand maturation of primary/secondary auditory cortex activity in typically-developing young children via assessing the maturation of the 50 ms (M50 MEG and P50/P1 EEG) response and the 100 ms auditory response (M100 MEG and N100/N1 EEG). M50 and M100 responses were examined in children 6–8 years old (Time 1) and again at ~18 months (Time 2) and 36 months (Time 3) after the initial exam. Given many studies showing hemisphere differences in the functional maturation of left and right auditory cortex activity (15, 27–33), analyses were conducted in source space (i.e., brain location), obtaining measures for left and right primary/secondary auditory cortex.

Based on cross-sectional findings (see Discussion), it was hypothesized that in each child M50 latency would decrease across the 3-year period and that M50 responses would peak earlier in the right than left hemisphere at all 3 time points (16, 34, 35). It was also hypothesized that in the majority of children M100 responses, as defined by a significant “peak” of detected power with opposite magnetic field topography occurring 50–100 ms later than the M50, would be observed only at Time 3 (36–38). Finally, qualitative assessment of Time 1 to Time 3 auditory cortex maturation (i.e., inspecting individual differences in maturation rate) was performed to assess between- and within-subject (between-hemisphere) rate-of-change variability in the M50 and M100 measures. It is hoped that findings will provide information that will benefit the design of pediatric auditory clinical studies (e.g., choice of age range to target given study goals).

## Methods and Materials

This study was approved by the local Institutional Review Board, and all families gave written informed consent. When competent to do so, children over 7 years of age gave verbal assent to participate.

### Participants

Data were obtained from 15 typically developing children (13M/2F; more males than females given that these controls were recruited for an autism spectrum disorder study). Children were selected according to the following criteria: (1) no history of traumatic brain injury, significant medical or neurological abnormality, known genetic syndrome, or diagnosed neurodevelopmental or learning disorders, (2) no active psychosis, (3) no MRI contraindications, (4) no sensory impairments (somatosensory, visual, or hearing), and (5) English as a first language. In addition, all children were evaluated by licensed clinical psychologists who ruled out the presence of DSM-5 Axis I disorders based on clinical judgment, review of parent-completed standardized, norm-referenced behavior rating scales, and parent screening interview (39–43). Finally, in all subjects, an estimate of full-scale IQ was obtained via a highly reliable 4-subtest short form (44) of the Wechsler Intelligence Scale for Children-5th edition (45).

### MEG Data Acquisition

MEG data were obtained using a 275-channel MEG system (VSM MedTech Inc., Coquitlam, BC). Electro-oculogram (EOG) (vertical EOG on the upper and lower left side) and electrocardiogram (ECG) were also obtained. After applying a band-pass filter (0.03–150 Hz), EOG, ECG, and MEG signals were digitized at 1,200 Hz with 3rd-order gradiometer environmental noise reduction. The participants' head position was monitored using three head position indicator (HPI) coils attached to the scalp. Children were scanned in a supine position.

The auditory exam consisted of sinusoidal tones of 500 Hz frequency and 300 ms duration. Tones were presented using E-Prime v1.1 via a sound pressure transducer and sound conduction tubing to the participant's peripheral auditory canal via ear-tip inserts (ER3A, Etymotic Research, IL, USA). Prior to each session, tones were presented binaurally and incrementally until reaching auditory threshold for each ear (i.e., stepwise approach). Tones were presented at 45 dB sensation level above threshold. Stimuli were presented with the inter-trial interval varying randomly between 600 and 2,000 ms, and with 520 trials collected over ~14 min. To minimize fatigue, during the task participants viewed a movie (without auditory track) projected onto a screen positioned at a comfortable viewing distance. After each MEG session, structural magnetic resonance imaging (sMRI) provided T1-weighted, 3-D MPRAGE anatomical images for source localization (3T Siemens Prisma scanner).

### Source Localization

MEG data were downsampled to 500 Hz. Artifact correction was applied to remove eye-blink activity using BESA 6.1, as outlined in Edgar et al. (14). Non-eye-blink artifacts trials were rejected by amplitude and gradient criteria (amplitude >1,200 fT/cm, gradients >800 fT/cm/sample). Artifact-free trials (from −500 ms to +500 ms) were then averaged.

Source localization for each subject was performed using their grand-average evoked response (e.g., collapsing across all ISIs). The average number of artifact-free trials was 477 at Time 1 (SD = 20), 470 at Time 2 (SD = 21), and 479 at Time 3 (SD = 20). To co-register MEG and sMRI data, three anatomical landmarks (nasion and right and left preauriculars) as well as an additional 200+ points on the scalp and face were digitized for each participant using the Probe Position Identification (PPI) System (Polhemus, Colchester, VT), and a transformation matrix that involved rotation and translation between the MEG and sMRI coordinate systems was obtained via a least-squares match of the PPI points to the surface of the scalp and face.

Left and right 50 ms (M50) and 100 ms (M100) sources were examined. As the primary generator of the M50 and M100 is well-modeled by a single dipole in left and right superior temporal gyrus (STG) and surrounding regions [(37, 46–49); see Edgar et al. (16) for an extended discussion on the generators of the M50 and M100 response], source localization was performed using an anatomical constraint. In particular, after co-registering the MEG and sMRI data, each child's left and right Heschl's Gyrus was visually identified and a dipole regional source [i.e., two orthogonal orientations (50)] manually placed at the “center” of each Heschl's Gyrus (i.e., at an anterior to posterior midpoint, and approximately two-thirds from the medial termination of Heschl's Gyrus; if two Heschl's Gyri were evident in a hemisphere, the dipole was placed between the two Heschl's Gyri).

After manually placing the left and right STG dipoles, the principle axis of each left- and right-hemisphere regional dipole source was oriented at the maximum of the left and right M50 and then left and right M100 response for each child in order to optimize the orientation of the standard regional sources (location fixed). Dipole orientations were obtained after applying a 2 Hz (24 dB/octave, zero-phase) to 55 Hz (48 dB/octave, zero-phase) band-pass filter to the scalp data used in source localization. Once oriented, the non-dominant source was removed and only the oriented source waveform examined. Goodness-of-fit (GOF = percent of sensor data explained) values for the M50 and M100 source models are provided in the Results.

Presence of an M50 and an M100 response was determined based on amplitude, latency, and hemisphere ingoing and outgoing flux topography. An M100 was scored if the magnetic flux topography was characteristic of the M100 response (i.e., for M100 left hemisphere ingoing anterior, outgoing posterior, and vice-versa for the right hemisphere), was preceded by M50 (i.e., flux topography opposite M100), and followed by M200 (i.e., flux topography same as M100), and with source strength greater than baseline. M50 was operationally defined as the first reversal in magnetic-field topography preceding M100 (or simply within a 35–125 ms range if M100 not present). In many children, a left- or right-hemisphere M100 response, defined as above, was not observed. For these children, left and right STG dipoles were oriented at the maximum of M200 [typically, M200 has a magnetic topography similar to M100 (16)]. When an M50 or M100 response was observed, left and right M50 (35–125 ms) and M100 (80–195 ms) scoring windows were used to identify the signal maxima in each window. This extended latency range allows capturing M50 and M100 responses in younger children.

### Statistical Analyses

As a left and right M50 response was observed in all participants, M50 latency was examined via an ANOVA with hemisphere and Time (1, 2, and 3) as repeated measures. Where appropriate, Huynh-Feldt corrections are reported. For the two participants unable to come to the Time 2 visit, the Time 2 M50 latency group mean was used to estimate their Time 2 M50 latency, and their M100 response was scored as missing. As left and right M100 were absent in the majority of participants, rather than examine M100 latency, the presence/absence of an M100 across hemisphere and time was examined via a Fisher's Exact Test and then follow-up McNemar analyses. Finally, correlations examined associations between age and M50 latency and between full-scale IQ and M50 latency, and Mann-Whitney *U*-tests examined associations between the presence/absence of an M100 and age and full-scale IQ.

## Results

### Demographics

Table 1 shows mean age and full-scale IQ at each time.

**Table 1:** Demographic information as well as left and right M50 (latency) and M100 (presence) values at each time.

	**Age**	**IQ**	**M50 latency**		**M100 present**	
	**Mean (SD)**	**Mean (SD)**	**Left mean (SD)**	**Right mean (SD)**	**Left**	**Right**
Time 1	7.83 years (0.70)	113 (13)	91 ms (13)	84 ms (10)	7%	40%
Time 2	9.23 years (0.70)	111 (14)	85 ms (14)	80 ms (8)	7%	40%
Time 3	10.82 years (0.70)	111 (10)	79 ms (12)	74 ms (5)	27%	67%

### M50 and M100 Recordings and Source Models

Motion during the recording was minimal, with average motion across the exam at Time 1 = 1.42 mm, Time 2 = 1.18 mm, and Time 3 = 0.95 mm. The lack of motion was largely due to the fact that the children were watching a video (with no sound track) during the exam.

Left and right M50 responses were observed in all recordings. GOF values indicated that the M50 source models (left and right oriented M50 dipole) accounted for a vast majority of the variance in M50 activity: Time 1 = 88%, Time 2 = 90%, and Time 3 = 88%. An ANOVA showed that M50 GOF values did not change as a function of Time, *F*_(2, 24)_ = 0.47, *p* > 0.05, and M50 GOF did not correlate with age (correlation values at each Time ranged from −0.34 to + 0.22, all non-significant).

As detailed in the following section, M100 responses were not observed in the majority of children. Examining only those individuals with a left, right, or bilateral M100 response, GOF values for the M100 source models were: Time 1 = 79%, Time 2 = 83%, and Time 3 = 79%. M100 GOF values were lower than M50 GOF values, in large part due to the fact that M100 responses were often very weak and also often only observed in a single hemisphere (see following section). Given very few children with an M100, change in M100 GOF across time and M100 GOF and age associations were not examined.

### Maturation of M50 and Associations With Age and IQ

Table 1 shows the left and right M50 mean latency at each time, and Table 2 left and right M50 latency for each child at each time (orange cells note the two children without Time 2 data, with their M50 latency values the group mean). ANOVA with Hemisphere and Time (1, 2, and 3) as repeated measures and M50 latency as the dependent variable showed main effects for Hemisphere [*F*_(1, 14)_ = 5.19, *p* < 0.05] and Time [*F*_(1.94, 27.18)_ = 16.24, *p* < 0.001], with M50 responses on average 5 ms earlier in the right than left hemisphere, and with M50 latency 10 ms earlier at Time 3 [77 ms (SD = 7)] than at Time 1 [87 ms (SD = 10); collapsing across hemisphere]. Neither age nor IQ was associated with M50 latency at any time point or with Time 1 to Time 3 M50 latency change (*ps* > 0.05).

**Table 2:** Left and right M50: latency for each child at each time (orange cells note the two children without Time 2 data, with their M50 latency values the group mean).

	**Age (years)**			**Left M50 latency (ms)**			**Right M50 latency (ms)**			**Left M100 0** **=** **no 1** **=** **yes**			**Right M100 0** **=** **no 1** **=** **yes**		
**Subject**	**Time 1**	**Time 2**	**Time 3**	**Time 1**	**Time 2**	**Time 3**	**Time 1**	**Time 2**	**Time 3**	**Time 1**	**Time 2**	**Time 3**	**Time 1**	**Time 2**	**Time 3**
L023	6.7	8.2	9.7	83	77	73	85	77	77	0	0	0	1	0	1
L024	6.7	8.1	9.8	91	75	79	83	73	75	0	0	0	1	1	1
L011	7.2	8.8	10.3	101	103	67	101	69	67	0	0	0	0	1	1
L006	7.3	8.9	10.3	79	73	71	83	81	83	0	0	1	1	0	0
L013	7.5	9	10.5	85	85	83	79	83	85	0	0	1	1	1	1
L010	7.6	9	10.5	83	77	73	81	75	69	0	0	0	0	0	1
L014	7.7	9.2	10.7	77	71	67	73	75	75	1	1	1	1	1	1
L032	7.8	Missed visit	10.8	93	85	81	81	80	75	0	0	0	0	0	0
L026	8.1	9.6	11.1	103	99	91	89	87	81	0	0	0	0	0	0
L015	8.2	9.7	11.2	97	89	81	79	77	75	0	0	0	0	0	1
L027	8.3	9.8	11.4	101	79	83	77	75	73	0	0	0	0	1	1
L018	8.3	9.8	11.3	103	105	93	107	101	75	0	0	0	0	0	0
L028	8.6	Missed visit	11.6	77	85	71	71	80	69	0	0	1	0	0	1
L007	8.7	10.2	11.9	115	111	107	89	89	71	0	0	0	0	0	0
L001	8.9	10.4	12	71	63	61	77	73	71	0	0	1	1	1	1

*Gray highlighting showing that whereas a M50 10ms latency reduction was observed in some participants from Time 1 to Time 2, this large latency reduction was observed in other participants from Time 2 to Time 3*.

**Figure 1:**
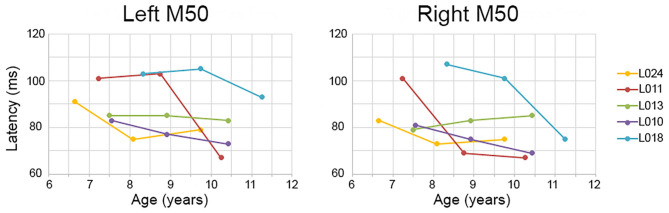
Examples of between- and within-subject variability in M50 latency change. Time 1, 2, and 3 (x axis) left and right M50 latency values (y axis) are plotted for five children. Whereas some children showed a constant M50 latency change from Time 1 to Time 2 to Time 3 (e.g., L010), in other children the M50 latency change occurred only between Time 1 and Time 2 (L011 right) or between Time 2 and Time 3 (L011 left).

Although the Time main effect confirmed the expected effect of an earlier M50 latency at Time 3 than Time 1 (i.e., statistically comparing Time 1 vs. Time 3 M50 latency), a qualitative review of the data showed that in many children, M50 latency changes were not constant between Time 1 to Time 2 and Time 2 to Time 3. This is detailed in Table 2, providing left and right M50 latency values for each participant at all three time points, with gray highlighting showing that whereas a 10 ms latency reduction was observed in some participants from Time 1 to Time 2, this large latency reduction was observed in other participants from Time 2 to Time 3. Also shown in Table 2, in some children M50 latency changes were consistent across the 3-year period, with a ~5 ms change from Time 1 to Time 2 and a ~5 ms change from Time 2 to Time 3. Finally, in some children (even the younger children) M50 latencies appeared to be adult-like across all three time points. Figure 1 plots left and right M50 latency values at each time point for five representative children (referred to in Discussion, and also see Supplementary Material for similar figure including all children).

### Maturation of M100 and Associations With Age and IQ

Table 1 shows the percentage of children with a left or right M100 at each time, and Table 2 the presence/absence of a left or right M100 for each child (orange cells note the two children without Time 2 data, with their M100 response scored as missing). Except for the right hemisphere at Time 3, an M100 was not present in the majority of children at any time. A Fisher's Exact Test examining the presence/absence of an M100 between hemisphere (2 levels) and across time (3 levels) was marginally significant (*p* = 0.07; the two participants with missing MEG data at Time 2 were scored as not having an M100). Simple-effects analyses, performed using the McNemar Test, showed that M100 responses tended to be observed more often in the right than left hemisphere at Time 1 (McNemar test *p* = 0.06) and Time 2 (McNemar test *p* = 0.06), but not Time 3 (McNemar test *p* = 0.13).

Mann-Whitney *U*-tests showed that the presence/absence of an M100 did not differ as a function of age or IQ at any time point. These analyses, however, were generally uninformative, as there were few children with an M100 response at Time 1, Time 2, or Time 3. Qualitative observations about the M100 are provided in the Discussion.

Finally, given two studies suggesting that in young children it might be difficult to observe M100 responses at short ISIs (31, 51), analyses examining the absence/presence of an M100 were rerun examining only trials with ISIs >1,600 ms (~120 trials). Although paired *t*-tests showed stronger M100 responses in the >1,600 ms ISI condition vs. shorter ISIs (*p* < 0.05; examining only those cases with a present M100), there was no evidence that M100s were more often observed at the longer vs. shorter ISIs. In particular, a review of all cases (15 children × 3 times) showed only two instances where an M100 would have been scored “present” when examining >1,600 ms ISI vs. scored “absent” when examining the “All” condition. A single child provided both instances (Time 2 right hemisphere and Time 3 right hemisphere).

## Discussion

Functional brain markers that can inform research on auditory processing abnormalities, and especially those ready to facilitate clinical work on such abnormalities, will need to show not only considerable sensitivity and specificity but enough consistency with respect to developmental course that their validity in individual cases can be trusted. A challenge to establishing such markers is individual difference in developmental course. Even in the present modest sample of typically developing children, large within- and between-subject variability was observed in the maturation of left and right primary/secondary auditory cortex function, a potential obstacle to establishment of useful auditory encoding markers in this age range.

Three findings were of note. First, although in almost all children M50 latencies decreased by ~10 ms across a 3-year period, the time course of this latency change varied across children. As an example, whereas in some children an M50 latency reduction occurred between Time 1 and Time 2, in others the M50 latency reduction occurred between Time 2 and Time 3. Second, the M100 response slowly developed across the 3-year period examined, with an M100 still not present in many children at their Time 3 visit (9–12 years old). Finally, and contributing to the large literature documenting hemisphere differences in auditory cortex activity, M50 and M100 findings underscored the need to examine left- and right-hemisphere auditory cortex neural activity separately (see Figure 1 and Table 2). In the following paragraphs, present findings are discussed with respect to previous studies examining the maturation of auditory cortex activity in children as well as with respect to the broader literature on brain markers in pediatric clinical populations.

With respect to M50, left and right M50 responses were observed in all subjects, a finding reported in many previous studies [e.g., see Ponton et al. (37)]. In young children, the P50 (EEG)/M50 (MEG) is readily evoked (52), with the peak latency of this component in 5- and 6-year-old children 85–95 ms (46, 53). These latency values are consistent with those observed in the present study (see Table 1, Time 1). P50 and M50 latency and amplitude decrease as a function of age (31, 54), with present findings indicating a ~10 ms change across the examined 3-year period. As noted, however, in many children this ~10 ms change was not constant across the 3-year period, with many different maturation scenarios observed. For example, as shown in Figure 1, in child L011 a large left M50 latency reduction was observed from Time 2 to Time 3 vs. a large right M50 latency reduction from Time 1 to Time 2. Many other patterns were observed; in child L018, left and right M50 latency changes were most prominent from Time 2 to Time 3, in child L010 a constant M50 latency reduction was observed across the 3-year interval, and in L013 the left and right M50 latency appeared stable across all three time points. Table 2 provides several additional examples of between- and within-subject variability in M50 latency rate of change.

Thus, despite a small sample of 15 children, present findings suggest a very wide variety of normal M50 development (assessed in terms of latency), including many younger children having earlier M50 latencies than older children (see Table 2). Although denser sampling, such as obtaining brain measures every 6 months, is needed to more exactly determine how rapidly M50 latency change can occur in this age range, the present data indicate an M50 latency “growth spurt” in many children, similar to how height can change rapidly in infants and children. Given the marked single time point as well as rate-of-change between-subject variability in M50 latency in this cohort (Time 1 age 6–8 years), “expected” associations between age and M50 latency were not observed, as changes in M50 latency in this cohort did not exceed the between-subject variability in M50 latency in this age range. Finally, and relatedly, previous research indicates that rate-of-change in M50 latency differs across the lifespan. As an example, examining cross-sectional infant data, Edgar et al. (30) estimated a ~0.6 ms/month latency change (= ~7.2 ms/year) for M50, with this rate-of-change estimate consistent with prior studies in this age range (55, 56). And, of course, in adults (young to middle-aged, pre-degenerative), M50 latency is expected to be stable.

**Figure 2:**
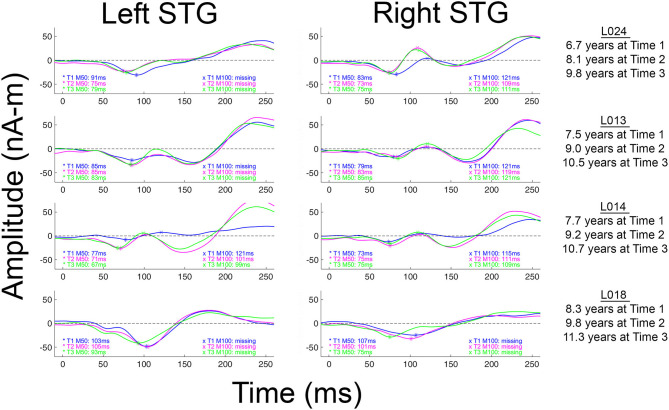
Left and right M100 source waveforms for all three times shown for four children. The x axis shows latency (ms) and the y axis source strength (nAm). When an M50 or M100 response was present, the peak latency value is provided. Children are ordered from youngest to oldest.

Although less common in young children, when present, N100 appears around 100–150 ms [e.g., (57–59)], with an adult morphology typically observed around 10–12 years of age (58), and thus with EEG N100 and MEG M100 auditory responses generally observed by late childhood and early adolescence (37, 58). Present findings showing missing left and right M100 responses in many of the children even at Time 3 (see Table 2), suggest that a N100/M100 response would not be observed in all children until early adolescence. Present findings, however, also demonstrated large variability in the absence/presence of M100 both between and within children. As an example, as shown in Figure 2 and Table 2, whereas an M100 response was observed in some children at all three time points (e.g., L024 in right and L014 bilaterally), many of the older children showed neither a left nor right M100 at any time (e.g., L018). And, analogous to M50 latency, within this age range, age was a poor predictor of the presence/absence of an M100 and the presence/absence of an M100 did not predict IQ, suggesting that variability in the presence/absence of an M100 in this age range is “normal.”

M50 and M100 maturation likely reflects changes to cortical gray matter such as changes in synaptic efficiency (32, 60) as well as maturational changes to the morphology of primary auditory cortex pyramidal cells (61, 62). Of note is that whereas development of deep layers (lower layer III to layer VI) in auditory cortex occurs between 6 months and 5 years of age (36), the superficial layers (upper layer III and layer II) continue to mature until about age 12 (63, 64). Based on this, researchers have hypothesized that the 50 ms auditory response reflects recurrent activation in layers III and IV, the termination zone of thalamo-cortical pathways that are almost fully developed by age 6 years. Observation of an M50 response in all children in the present study is consistent with early development of cortical layers III and IV, with changes to M50 latency likely reflecting, in part, continued maturation of thalamo-cortical white matter (e.g., myelination) through childhood and early adolescence (65, 66). As the M50 and P50 EEG response is observed in infants (30, 53, 67–69), the M50 response appears to be present throughout the lifespan and thus can be tracked from infancy through adulthood [e.g, see Figure 3 in Chen et al. (26)]. To the extent that M50 primarily reflects activity from cortical layers III and IV, the M50 response thus allows assessment of the maturation of these cortical layers across the lifespan.

As previously noted, M100 is observed less frequently in young children. It has been hypothesized that this is due to the fact that generation of M100 likely reflects activation of cortical layers upper III and II, areas not fully developed until at least age 12 [e.g., (36, 37, 58)]. In Figure 2 (and see all source waveforms plots in Supplementary Material), the gradual emergence of an M100 response is observed, with the slow development of M100 perhaps reflecting greater synchronization in the afferent activity arriving at the synapses in layer II and upper layer III (38) across childhood.

Maturational changes to auditory cortex function, as assessed via the use of electrophysiology, thus likely occur within a landscape of altering feedforward and feedback inputs to primary/secondary auditory areas. Ponton et al. (37) suggested that during development, the magnitude of the earlier maturing tangential “50 ms” auditory response decreases as the magnitude of the later maturing tangential “100 ms” auditory response increases. Given this pattern, in the present study, a developing M100 response could result in an earlier M50 response via “cancelation” of M50 activity via an increasingly dominant M100 response.

Whereas, in young children M50 activity may “cancel” M100 activity, it is also hypothesized that in older children the feedback activity giving rise to the M100 response increases in strength, such that in adults the neural activity associated with M100 is strong enough to largely “cancel” the M50 response. As suggested by Ponton et al. (37) and Ceponiene et al. (46), what is possible is that rather than M50 decreasing as a function of age, the M50 response simply appears smaller in EEG and MEG recordings given cancelation of an external M50/P50 response by stronger electrical currents associated with the M100 response. Of course, another possibility is that the M50 response truly becomes weaker as a function of age. Although corticography studies could help differentiate between these two alternatives, such studies will be difficult given the need to study sulcal auditory cortex neural activity in children at different ages.

In several papers, including a paper by our group, it has been suggested that the M100 develops “out of” the later N200 (EEG) or M200 (MEG) response (16, 36, 37). The N200/M200, a response occurring after N100/M100 and with the same topography as N100/M100, has a maximum amplitude at ~8 years and then decays until it is often not present in individuals 18 years and older (37, 70). In Figure 2 (and see Supplementary Material), the M200 response is clearly observed in all children. Present findings, however, do not support the claim that the M100 develops out of the M200. Indeed, as shown in the Figure 2 and the Supplementary Material source waveforms, in most children the M100 appears as a distinct component, slowly emerging in-between M50 and M200. As a specific example, in L013 (Figure 2), the “M100” is at all times clearly distinct from the later M200.

**Figure 3:**
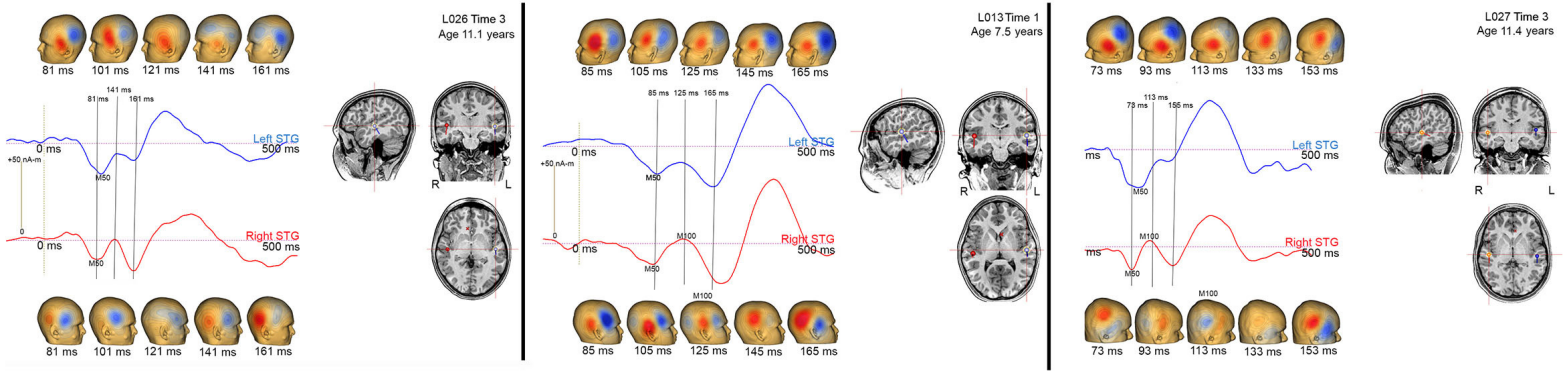
Left (blue) and right (red) STG source waveforms (−75 to 500 ms), magnetic field maps (20 ms intervals), and left and right Heschl's gyri dipole locations for 3 children. Data are presented to provide examples of the criteria used to determine an absent/present M50 and M100.

In the present study, M100 was scored as present if there was a peak with a rising and falling slope distinct from M200, with an M100 magnetic-field topography, with a non-zero source strength, and with a latency between 80 and 185 ms. Figure 3 shows examples of how the study scoring criteria were applied to determine the absence/presence of an M100. Specifically, Figure 3 shows left and right STG source waveforms (−75–500 ms), magnetic field maps (20 ms intervals), and left and right Heschl's gyri dipole locations for three children. In all three children, M50 responses were observed bilaterally (M50 responses are plotted “downward”), with the source waveforms showing an M50 response that exceeds baseline and the magnetic field maps showing the expected M50 field pattern (e.g., in the left hemisphere an anterior magnetic field source and a posterior magnetic field sink). None of the three children showed a left M100 response, mirroring what was observed in the vast majority of the sample. And of note, although in all three children a left M100 is suggested, in none of the children did the left magnetic field maps indicate an M50 to M100 field pattern reversal, and thus a left M100 was scored as absent in all three children. In contrast in children L013V1 and L027V3, a right M100 was scored as present. In L013V1, although a full field reversal from M50 to M100 is not observed, the magnetic field pattern clearly indicates a field reversal (although not as purely dipolar as an adult M100) and the M100 response exceeded the baseline (barely), and thus an M100 was scored as present. In L027V3, a more mature right M100 is observed, with an M100 magnetic field pattern clearly observed and M100 weak but clearly exceeding the baseline. These three children provide examples where researchers using different scoring criteria, for example not requiring a magnetic field reversal from M50 to M100, might have scored M100s as present (e.g., scoring based on examining only the source waveforms or only the EEG or MEG sensor responses). Notably, these three cases differ from the many other children where there was no source waveform deflection suggesting an emerging M100 (e.g., see L018V1).

Some researchers may regard the M100 scoring criteria applied in this study as too conservative; although in many children M100 was scored as missing (e.g., an M100 magnetic field topography was not observed), what is likely the “M100” response could be observed in the source waveforms at Time 1, Time 2, and Time 3 (e.g., L013 left in Figure 2). Although conservative, this is a safe strategy, as the longitudinal data indicate risks associated with a more liberal approach. In particular, the present three-time-point data likely provide a false sense of security; although in this study the Time 3 data in many children often provided visual confirmation that a Time 1 or Time 2 M100 could be identified based only on source waveform morphology (e.g., L013 left in Figure 2), in most studies, investigators do not have the ability to look into the future to determine whether they made the right choice.

Difficulty scoring the M100 in this age range is of note when examining the multi-determined sensor data rather than source waveforms. Indeed, present findings indicate that developmental studies examining N100 activity at a single midline scalp site (e.g., Cz or Fz) are problematic, as latency and amplitude measures at a single site in many children will reflect activity from only a single hemisphere (e.g., see L024 in Figure 2). Without separately examining left and right auditory cortex activity, it is not possible to determine which hemisphere or perhaps even which components (M100 or M200) contribute to the midline EEG N100 response.

Other study findings also demonstrated the need to separately examine left and right auditory cortex activity. An example is the left vs. right STG M50 latency difference observed in the present study, a finding reported in previous studies examining children and adolescents (15, 16, 27–29). Given left and right M50 latency differences in pediatric populations, examination of the latency of the 50 ms latency response at EEG midline sites would be problematic. More generally, Ponton et al. (37) and Sussman et al. (71) have noted that examining sensor auditory data is problematic as the activity at any given sensor location reflects the weighted contribution of activity from different sources, each with potentially different maturation rates. The Edgar et al. (72) left and right auditory cortex simulations provide a detailed examination of the problems with scoring auditory cortex activity at the sensor level, with hemisphere differences in the latency or amplitude of an auditory response providing very misleading EEG midline auditory measures. In addition to the above concerns, an analysis strategy that provides separate measures for left and right auditory activity is critical in pediatric clinical studies as studies show hemisphere-specific abnormalities in neurodevelopmental disorders [e.g., see (15, 73)].

Considered as a whole, the present M50 and M100 findings indicate that variability in the maturation of auditory cortex neural activity occurs in a manner analogous to maturation of behavioral phenotypes observed “by eye.” For example, although most typically developing infants take their first steps between 9 and 12 months and are walking by 14 or 15 months, some normally developing children do not take their first steps until 16 to 17 months (74). Furthermore, within this almost 1-year range, age of first step does not predict future intelligence or coordination (75). Only by 20 months does an infant who does not walk become of clinical concern (76, 77).

As observable “by eye” behaviors relate in some way to brain activity, it is thus perhaps not unexpected that the relatively large between-subject variability observed in behaviors that develop during infancy and childhood (walking, talking, skipping) is also observed in brain measures. In the present study, large between- as well as within-subject (between-hemisphere) variability was observed in the maturation of M50 (latency change), with the M50 and M100 measures not related to age at time of first exam or to general cognitive ability (IQ). The slow development of auditory cortex neural activity is perhaps is related to the slow process of language development, extending from birth to teenage years. For example, although children have essentially mastered the phonology of their language by 5 years of age, their articulation continues to develop as they start to use more complex stress and intonations (78). Regarding speech perception, their ability to understand speech in noisy circumstances continues to improve up to age 15 (79).

As detailed in Edgar et al. (22), an understanding of normal brain maturation is needed whether one takes a DSM-5 or an RDoC approach to research. What differentiates these approaches is primarily a matter of granularity (80), with a DSM-5 approach focusing on diagnosis and an RDoC approach focusing on smaller units of analysis that may cut across DSM-5 diagnostic categories, such as psychological concepts and biological phenomena associated with disease (80, 81). The present study provides specific examples of potential RDoC biological phenomena—M50 and M100—and demonstrate the need to consider hemisphere differences (M50 and M100) and also demonstrate that some measures are of use only after a certain age (M100).

**Figure 4:**
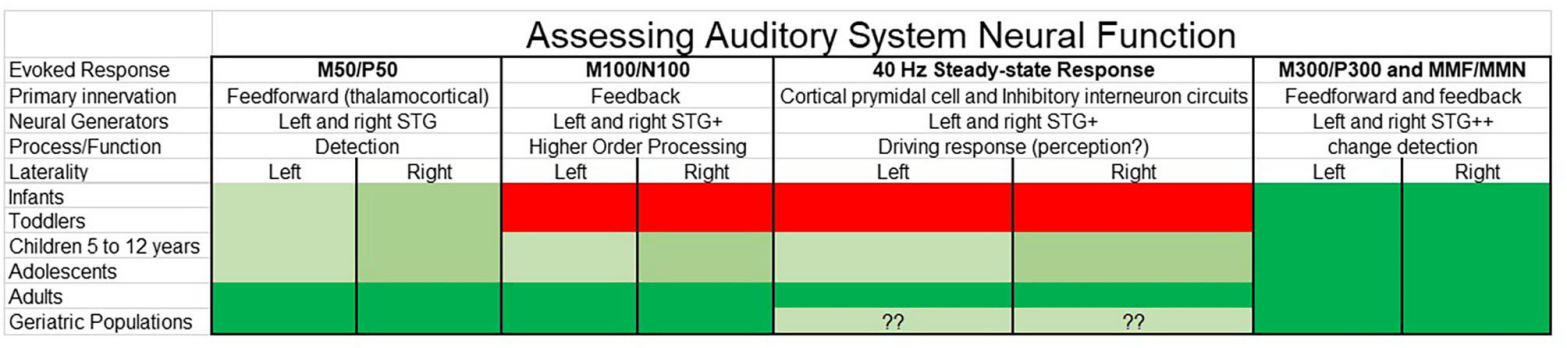
Auditory system neural measures for clinical research considered as a function of the human lifespan (green = possible, red = not possible) as well as with respect to neural and cognitive functions of interest. Although not comprehensive, relevant features to consider are noted, such as the likely brain regions involved, and with hemisphere differences in rate of maturation [hemisphere showing slower maturation (lighter green) compared to hemisphere showing faster maturation (deeper green)]. Whereas research indicates that some auditory evoked responses such as M50 and P50 can be measured throughout the lifespan, other measures such as the M100 and N100 and the auditory 40 Hz steady-state response are reliably obtained only in older adolescents and adults. Also of note are differences in the “complexity” of the evoked response—whereas some evoked responses such as M50 and P50 are thought to primarily reflect activity from left and right primary/secondary auditory regions, other evoked responses reflect activity from multiple brain regions, such as the M300 and P300 (and with almost no research examining rates of maturation for each M300 and P300 generator).

Presented in a “RDoC format,” Figure 4 suggests a way to conceptualize assessment of auditory system neural function, with the selection of auditory measures of interest considered with respect to what neural and cognitive functions are of clinical interest as well as what research tells us so far about the presence/absence of specific auditory components across the lifespan. Although not comprehensive, Figure 4 sketches out some of the relevant features to consider [including noting hemisphere differences in rate of maturation via lighter (slower) to deeper (faster) shades of green]. Although scientists might disagree with some of the details, such disagreements simply indicate that there is more to learn. And although the present study focuses on electrophysiology auditory cortex measures, as detailed in Edgar (22), these concerns are relevant to other imaging modalities and other ages.

With respect to identifying clinical auditory neural brain markers (e.g., brain measures diagnostic of autism spectrum disorder or learning disability), the present longitudinal findings also provide some perspective. For example, although it is likely that control and patient group differences in M50 latency can be detected with a moderate-sized sample [see Edgar et al. (16) for an example in autism spectrum disorder], examination of the present longitudinal data suggests that it is unlikely that an M50 latency diagnostic marker with high sensitivity (correctly identifying all patients) and specificity (not mistakenly identifying a control as a patient) could be developed. This is because it is doubtful that in a given patient group all the patient's auditory measures would exist at the tail of the control distribution (the state of affairs needed to obtain high sensitivity and specificity). As a specific example, using right M50 latency as a marker in the clinic for children 6–8 years old, and given the Time 1 right M50 latency standard deviation of 10 ms (mean = 84 ms; range = 71–107 ms), almost all members of the patient group would need to have a latency great than 104 ms (i.e., showing delayed M50 latencies) to obtain high sensitivity and specificity. The above example simply provides another example of what functional and structural brain imaging clinical research findings already show. In particular, in most neurodevelopmental disorders a single brain marker has not been shown to have the sensitivity and specificity needed to serve as a diagnostic brain marker, this claim supported by the fact that large sample studies show at most small to medium group effects for most brain measures [e.g., (82–84)].

Of course, single auditory measures may prove useful in other ways, such as predicting treatment response (85). And as evidenced in other papers, multiple brain measures can be combined to provide greater group separation (86). Such composite diagnostic measures, however, may be difficult to obtain given that different brain measures may provide optimal group separation at different ages [see Edgar et al. (22) for a discussion].

A few study limitations are of note. First, findings are generally specific to right-handed typically developing males (only two female participants), with studies needed to determine whether there are sex differences in the maturation of auditory cortex (from birth to late adolescence). Second, larger samples (males and females) are needed to determine what is normal vs. what is atypical, similar to the growth charts available for height and weight (e.g., 95% confidence intervals for a given age). However, although larger samples are needed to fully characterize M50 and M100 auditory cortex development, the present sample is sufficient (and sufficiently well-screened) to demonstrate that large variability across typically developing children in the maturation of auditory cortex is typical, just as similarly large variability in age of first step is typical. Third, as study participants have not yet been followed into adolescence, it is unknown at what age all participants will show a left and right M100 response. Fourth, the present study focused on evoked components, with future studies examining maturation of time-frequency measures (e.g., intra-trial coherence, event-related synchronization/desynchronization) certainly of interest in neurodevelopmental patient populations [e.g., (14, 87–91)]. Finally, the present study did not examine associations between maturation of brain function and brain structure (e.g., local gray matter). Such function-structure analyses will be conducted once the samples are much larger.

In conclusion, the present paper examined within- and between-subject variability in the development of auditory cortex neural activity in children. Relatively large between-subject as well as within-subject (left- and right-hemisphere) variability in reaching neural developmental milestones (e.g., showing an M100 response) was observed. Findings also clearly demonstrated the need to examine whole-brain activity given regional differences (e.g., hemisphere) in the rate of brain maturation.

## Data Availability Statement

The datasets presented in this article are not readily available because data will be made available upon request at the conclusion of this longitudinal study. Requests to access the datasets should be directed to edgarj@chop.edu.

## Ethics Statement

This study was approved by the Institutional Review Board at the Children's Hospital of Philadelphia and all families gave written informed consent. Written informed consent to participate in this study was provided by the participants' legal guardian/next of kin.

## Author Contributions

LBla, KK, EM, EK, MA, and MKi: individuals contributed via developing and administering clinical assessments. JCE, HG, GS, MD, JB, LBlo, SL, and MKu: analysis of MEG data. JCE, LBlo, RF, GM, and TR: statistical analysis. All authors were involved in writing and editing the manuscript.

## Conflict of Interest

JB reports a consultancy with McGowan Associates. TR declares his position on the advisory boards of (1) CTF MEG, (2) Ricoh, (3) Spago Nano Medicine, (4) Avexis Inc., (5) Acadia Pharmaceuticals and equity interests in (1) Prism Clinical Imaging and (2) Proteus Neurodynamics. TR and JCE also declare intellectual property relating to the potential use of electrophysiological markers for treatment planning in clinical ASD. LBla reports a consultancy with KBT Technologies. The remaining authors declare that the research was conducted in the absence of any commercial or financial relationships that could be construed as a potential conflict of interest.
