# Supplementary figures and images for: Maturation of Auditory Cortex Neural Activity in Children and Implications for Auditory Clinical Markers in Diagnosis
Source: Front Psychiatry. 2020 Nov 19;11:584557. doi: 10.3389/fpsyt.2020.584557 (PMC7717950; doi:10.3389/fpsyt.2020.584557)

Figure 2

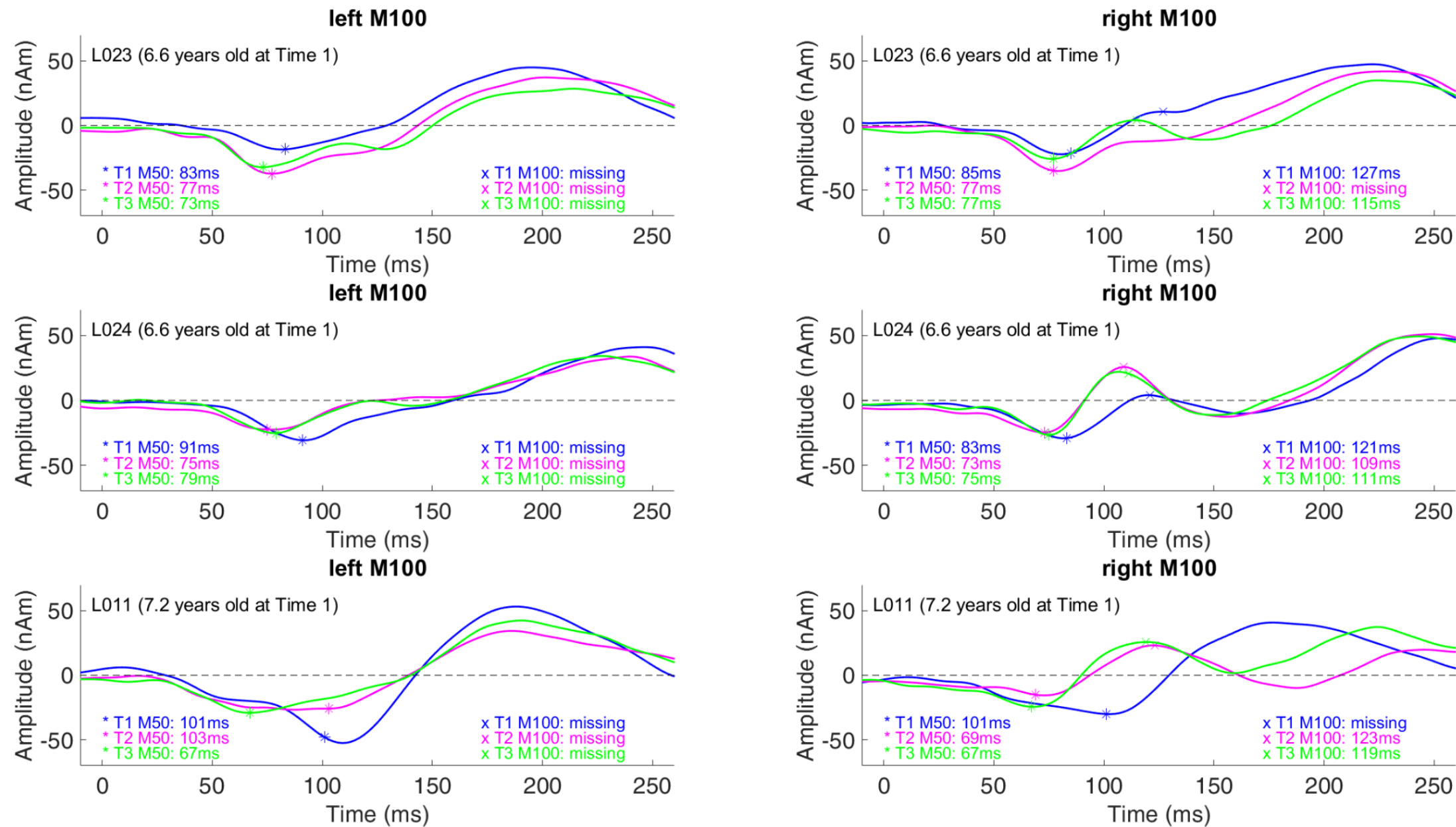

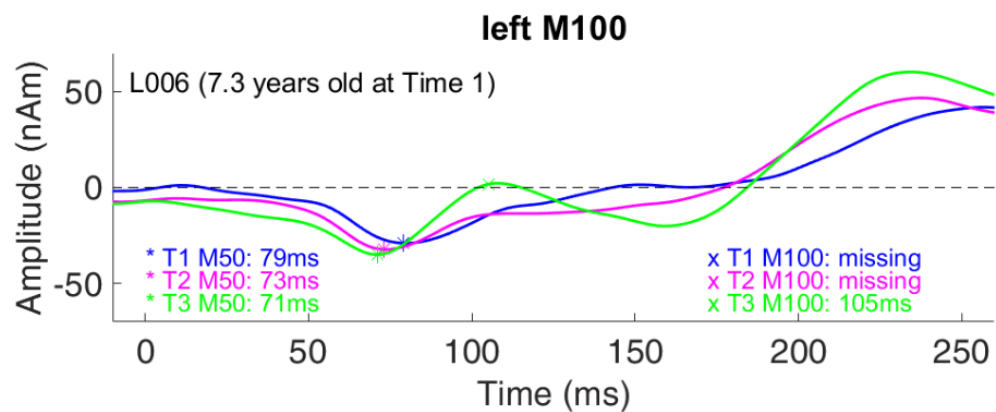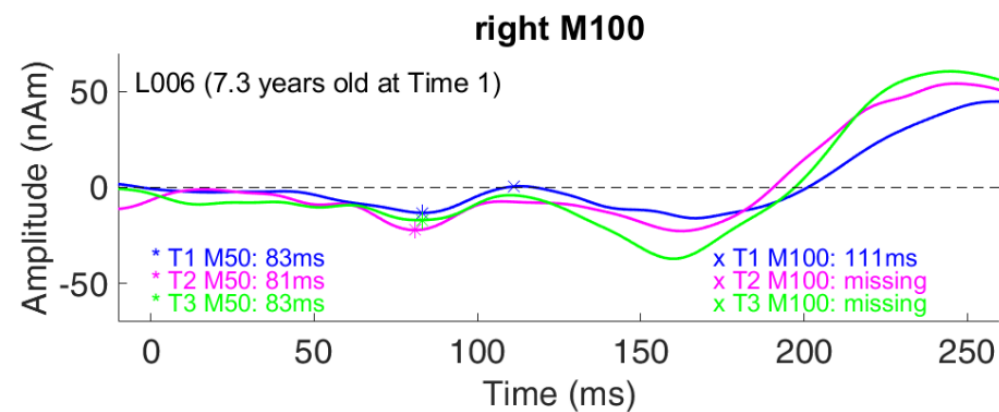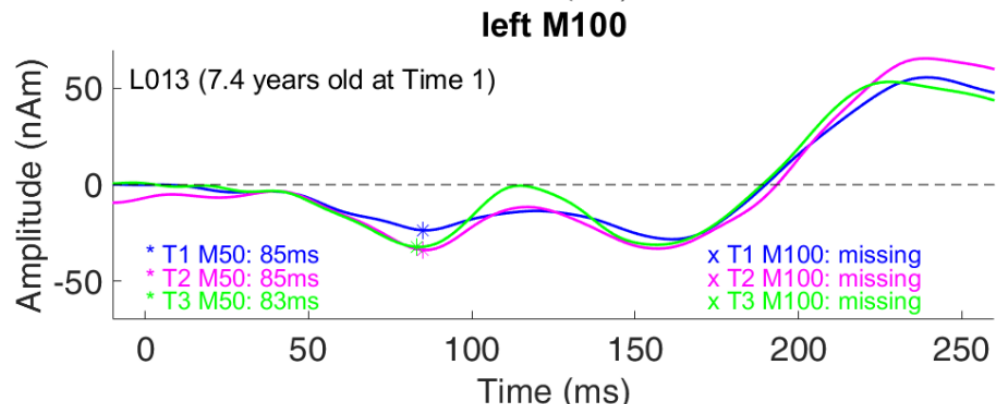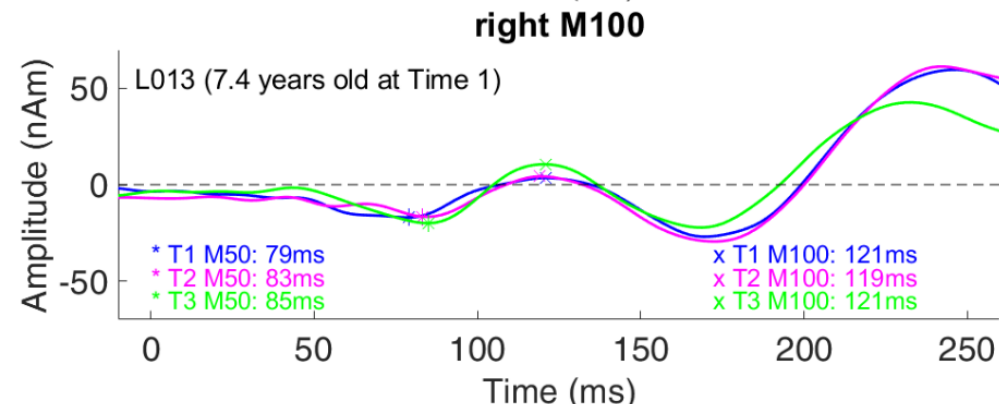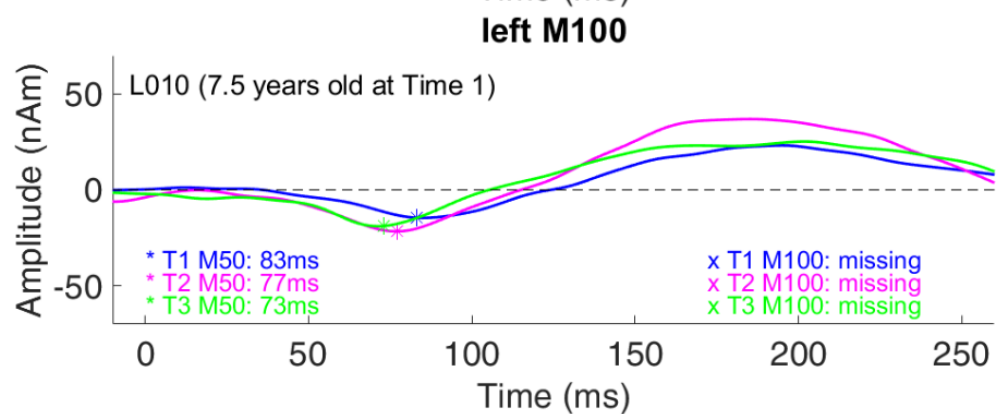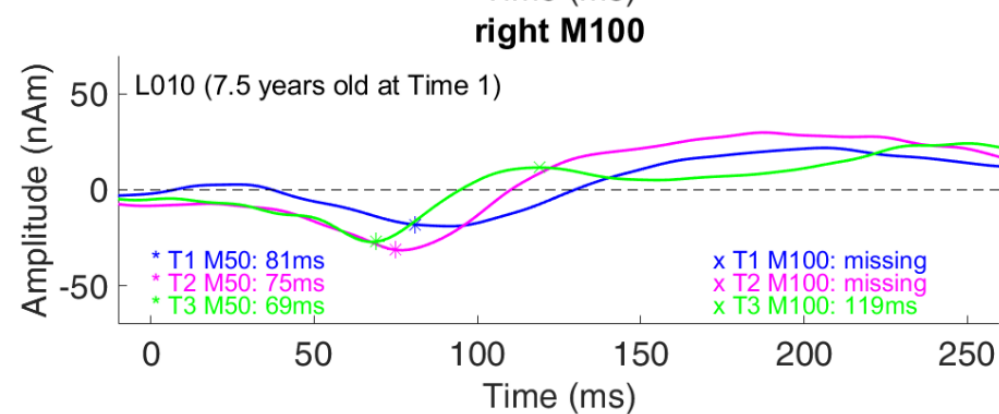

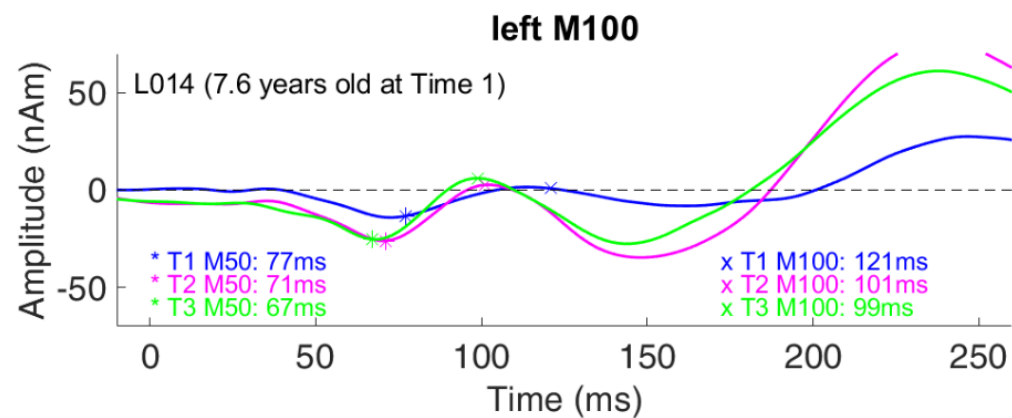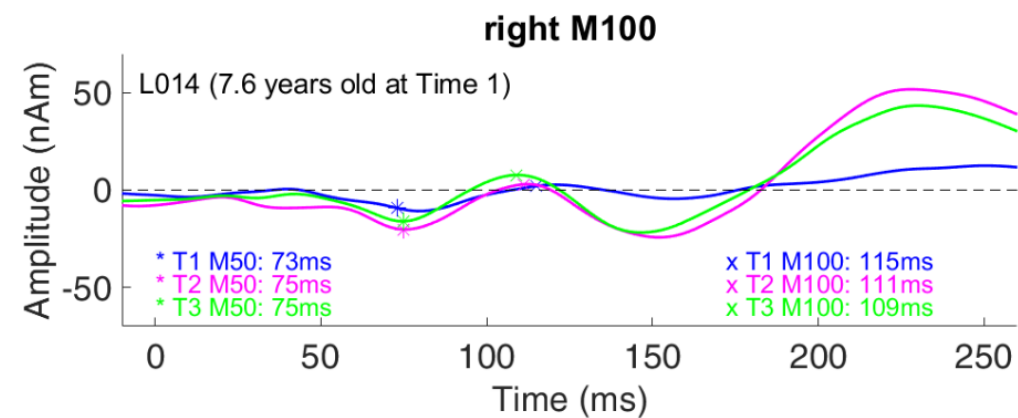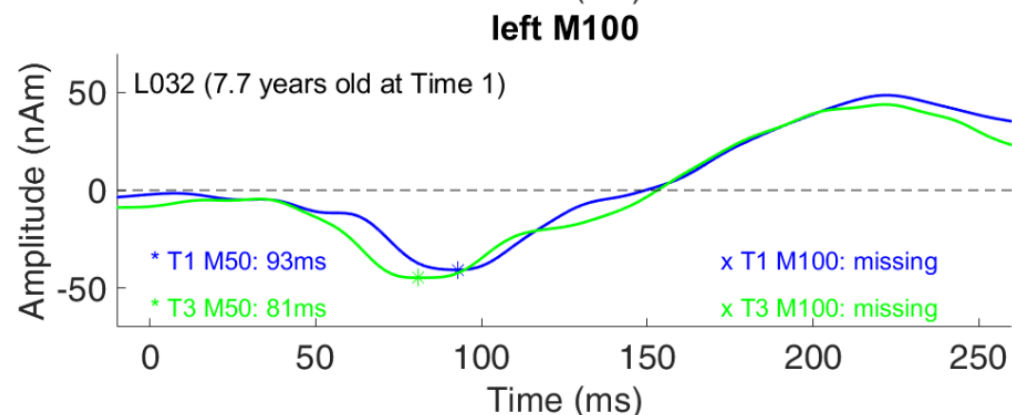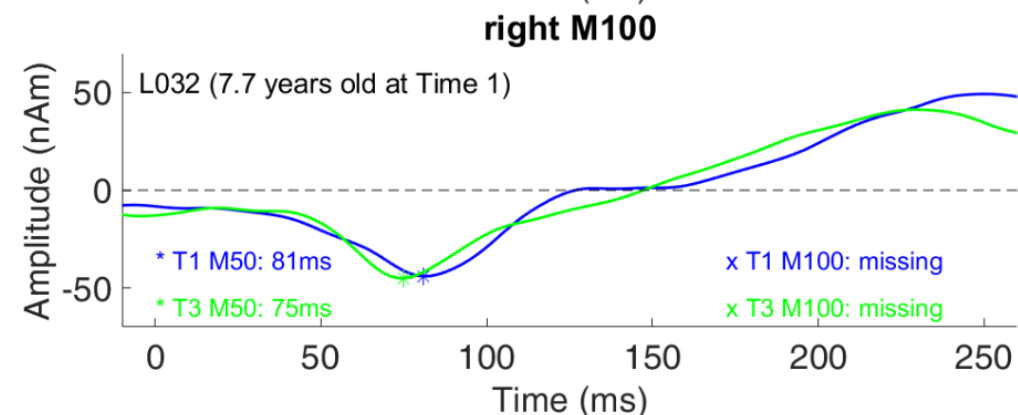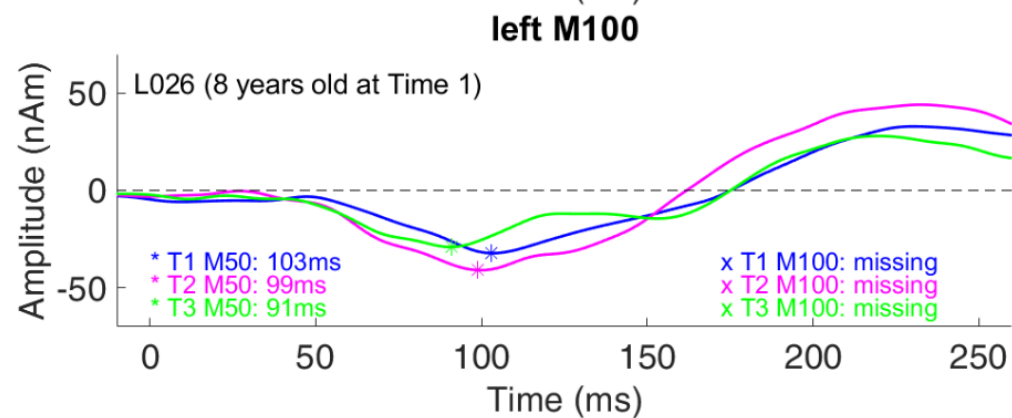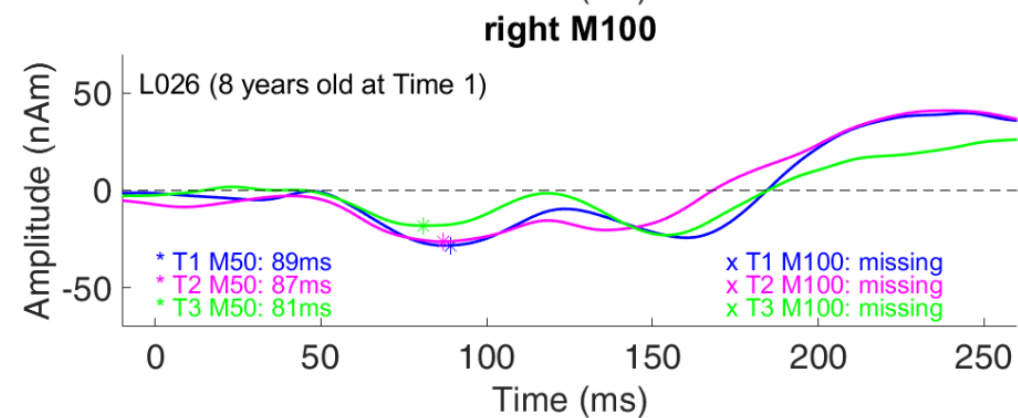

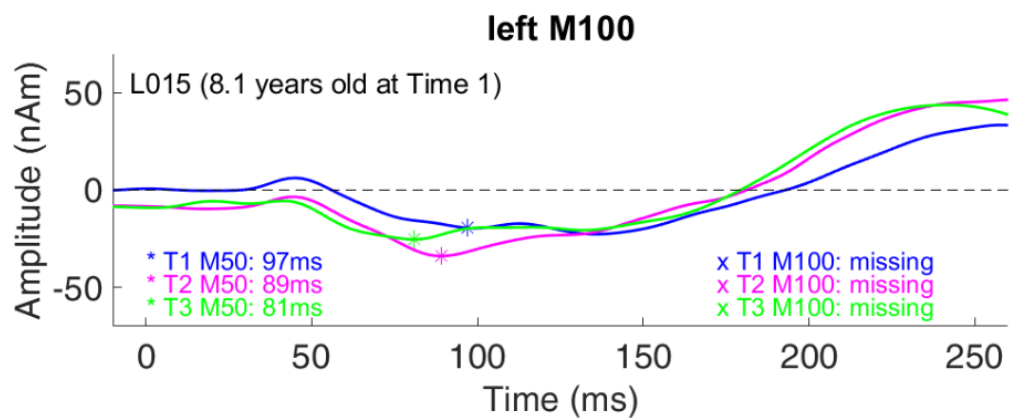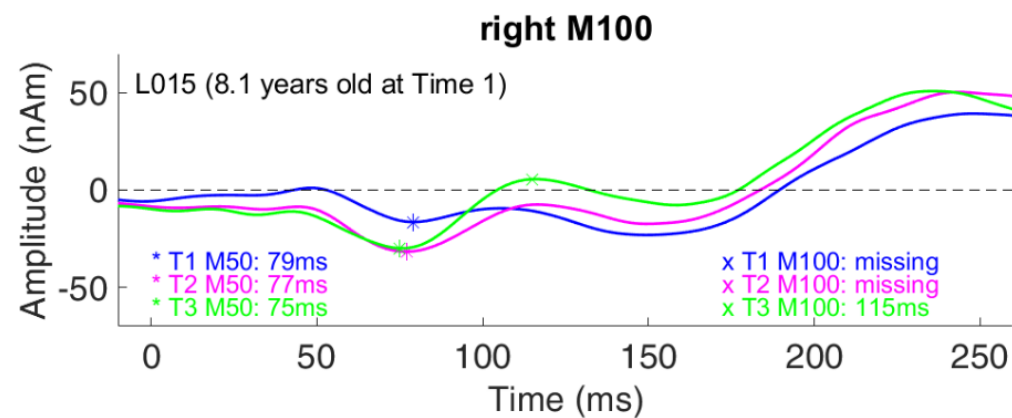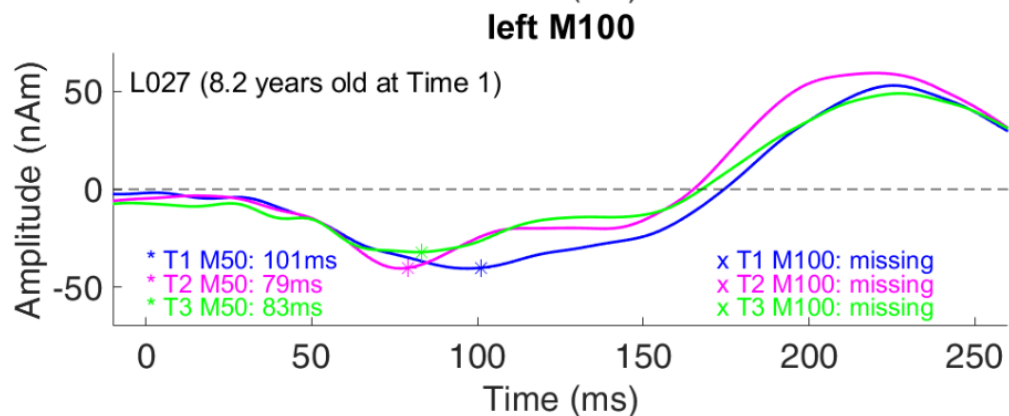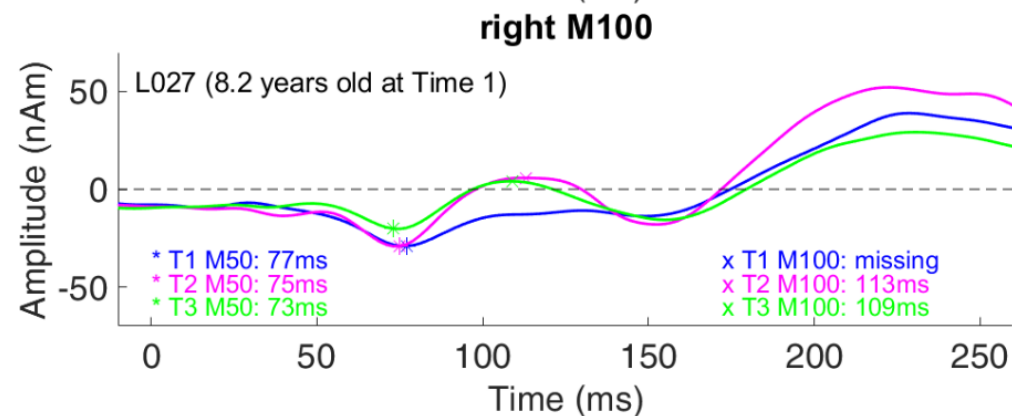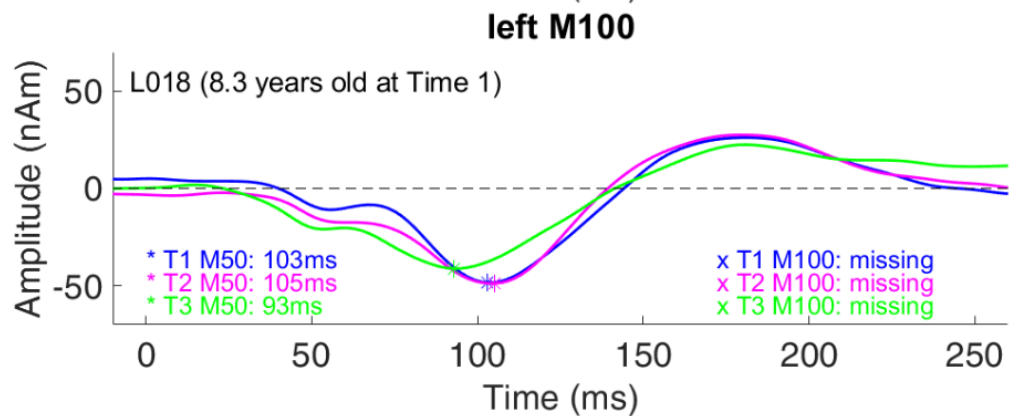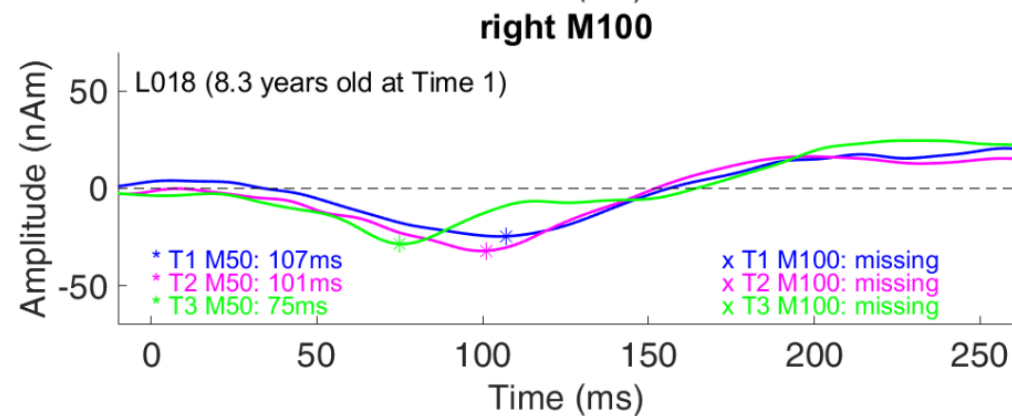

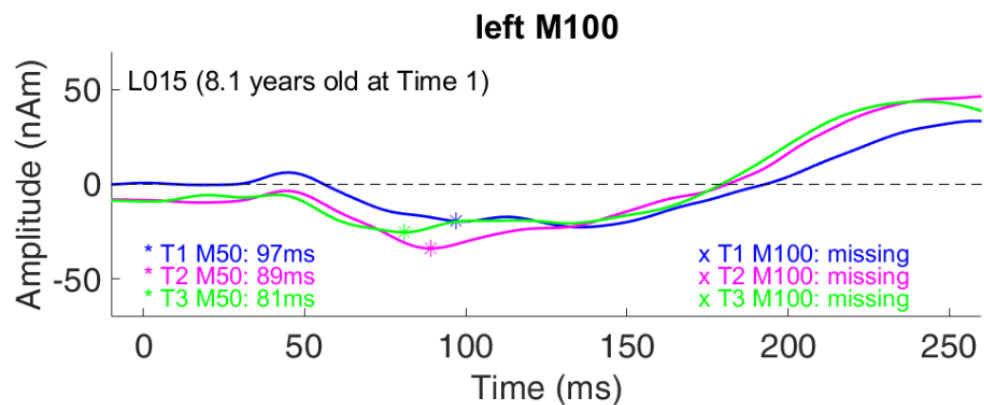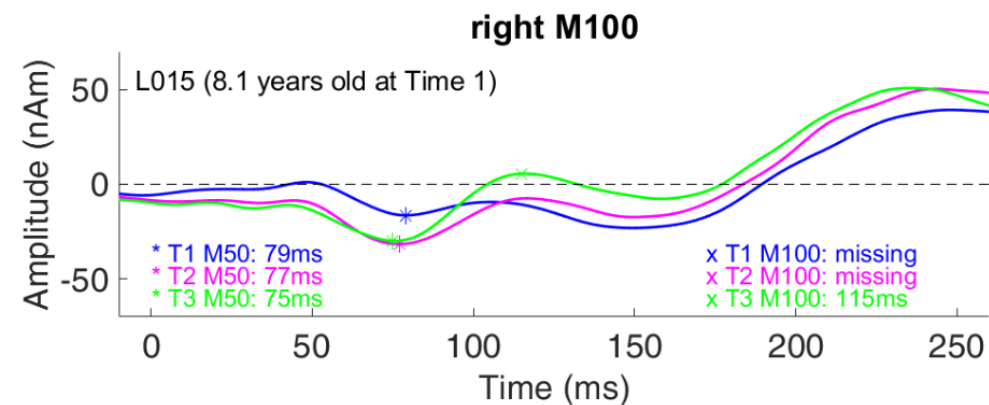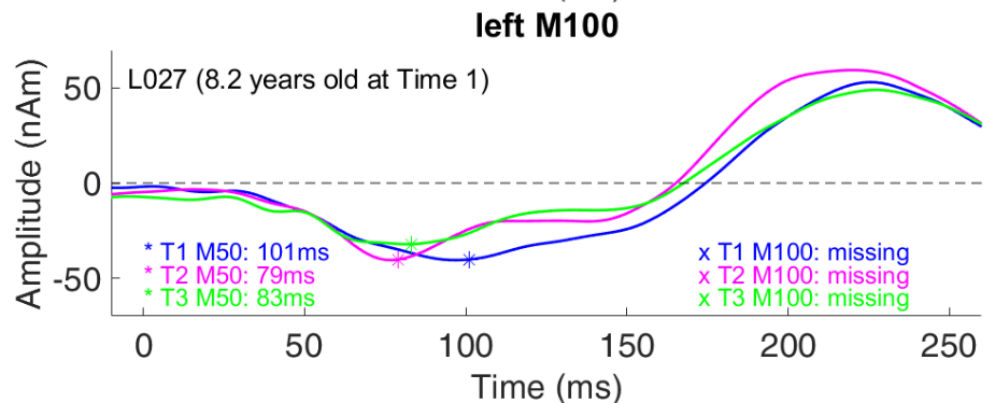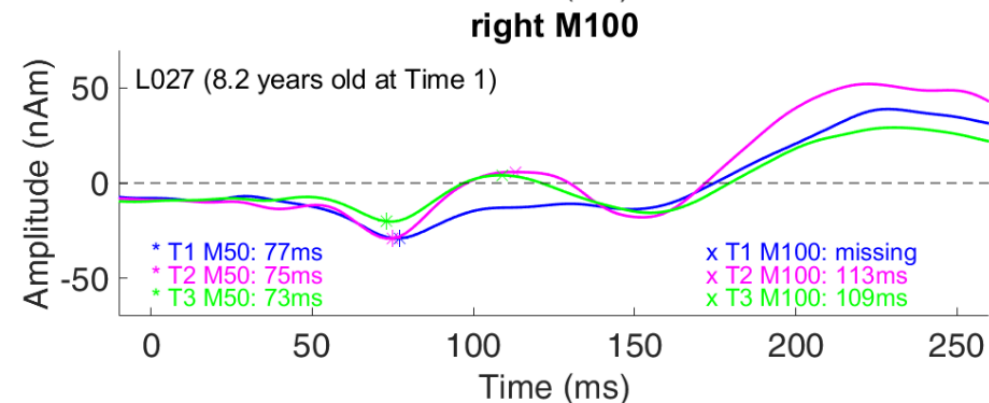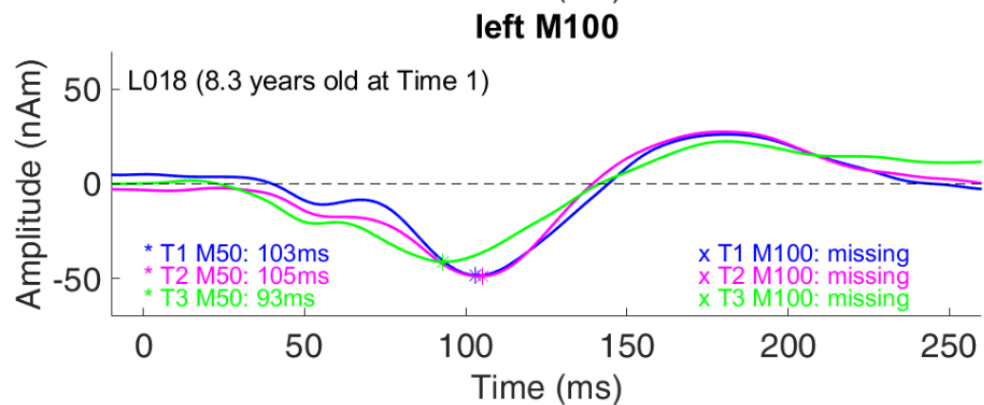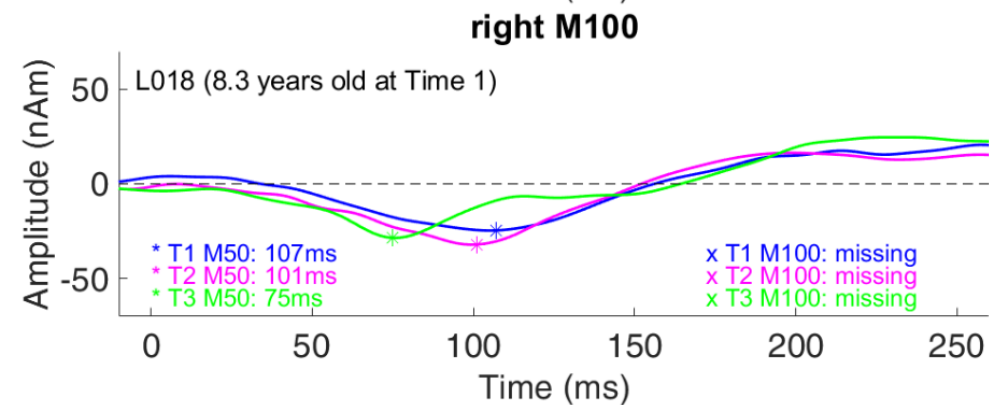

Supplement: Supplementary file 1 [file Data_Sheet_1.PDF]

Figure 3

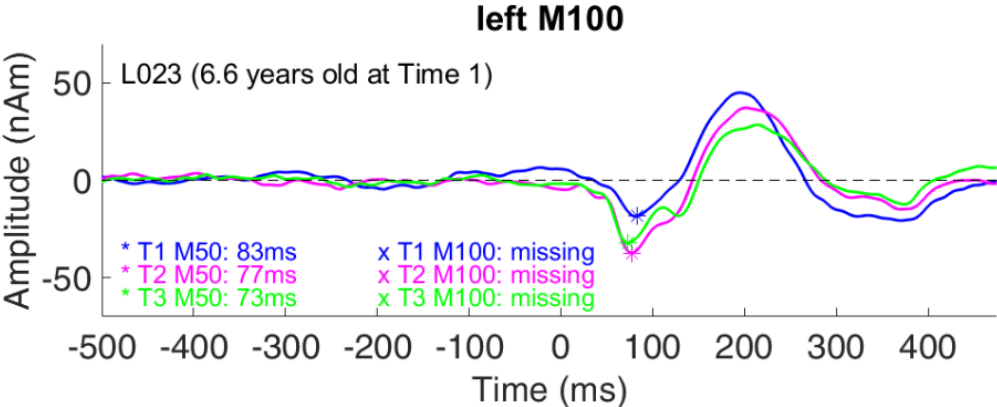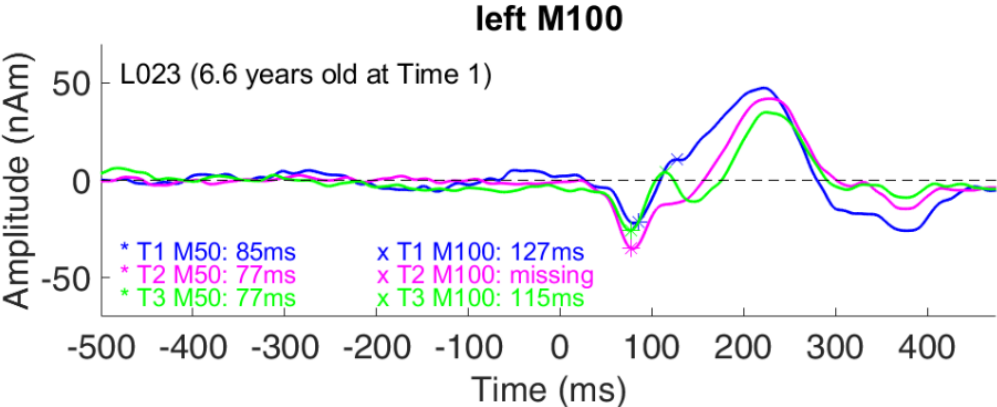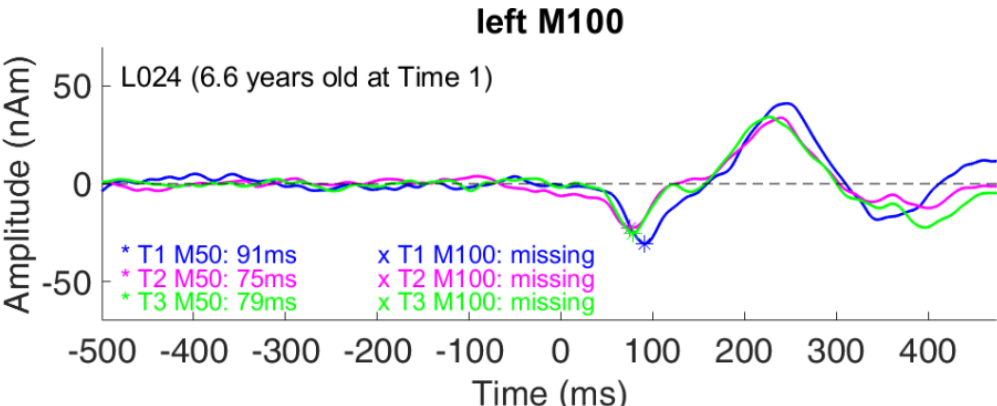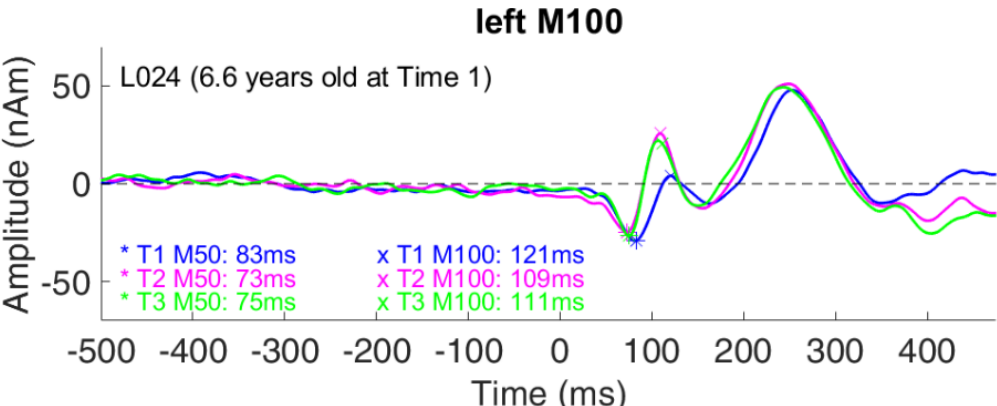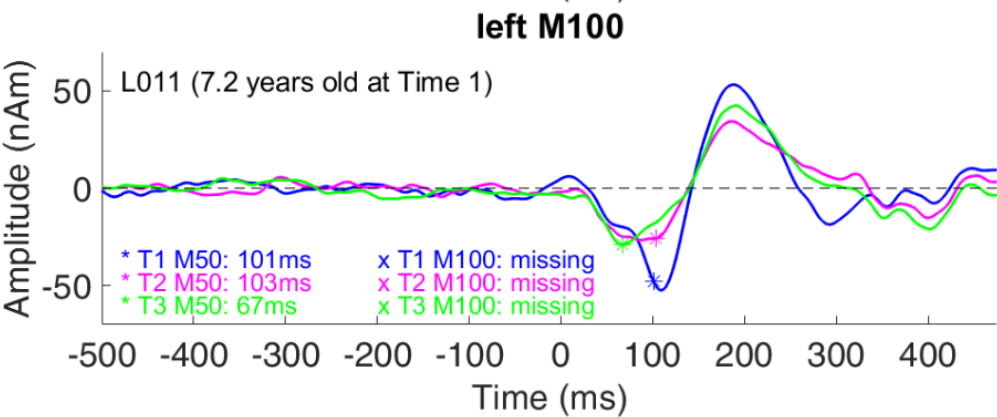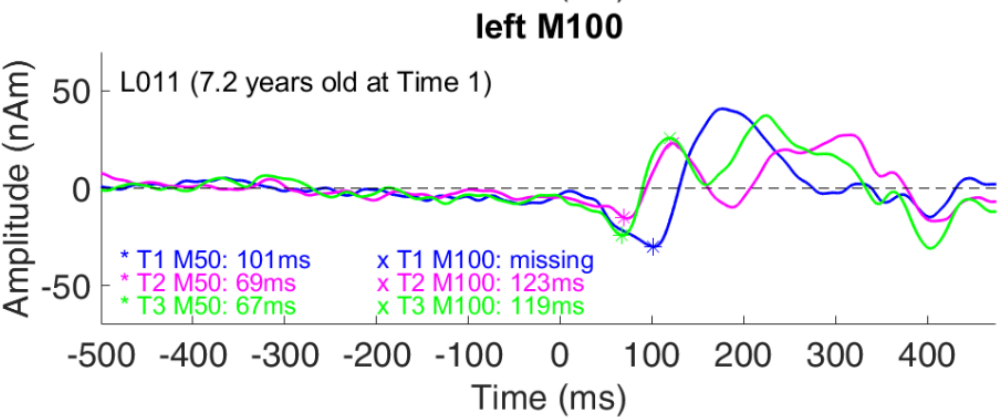

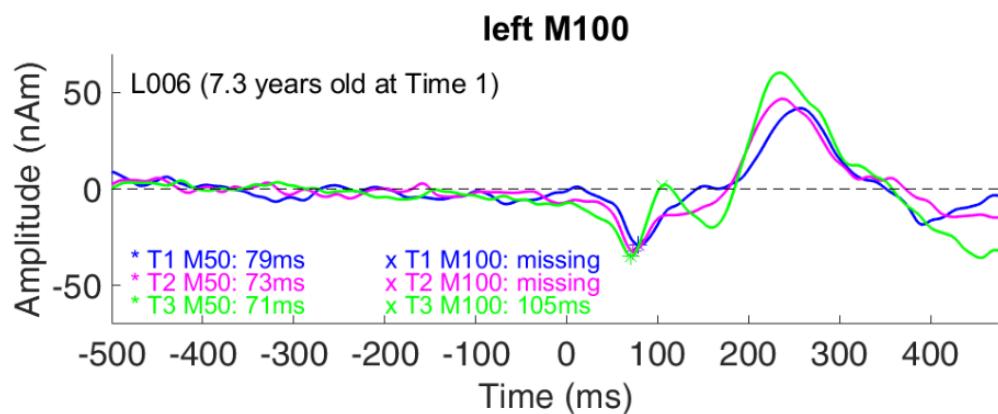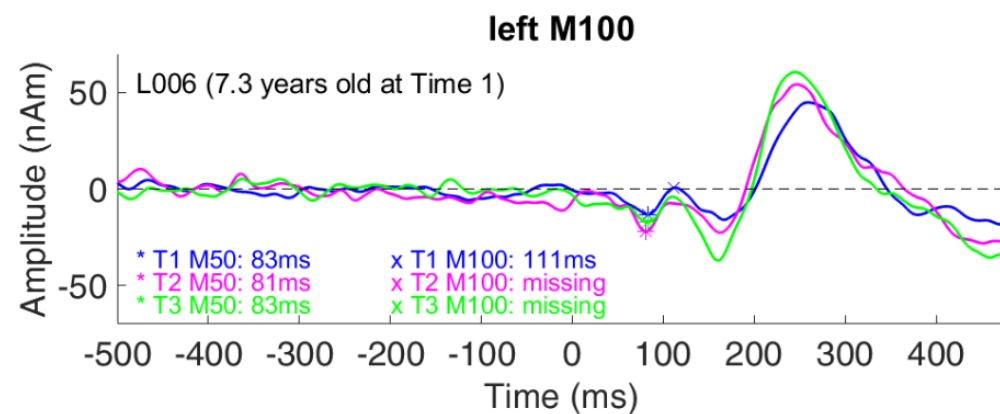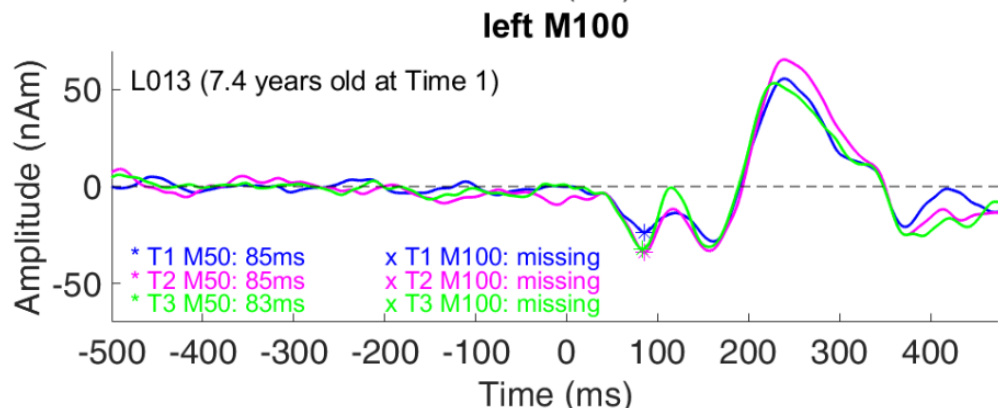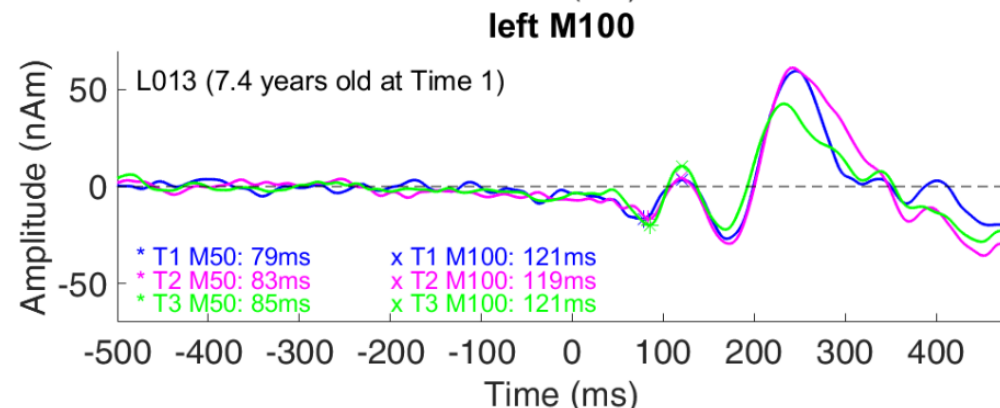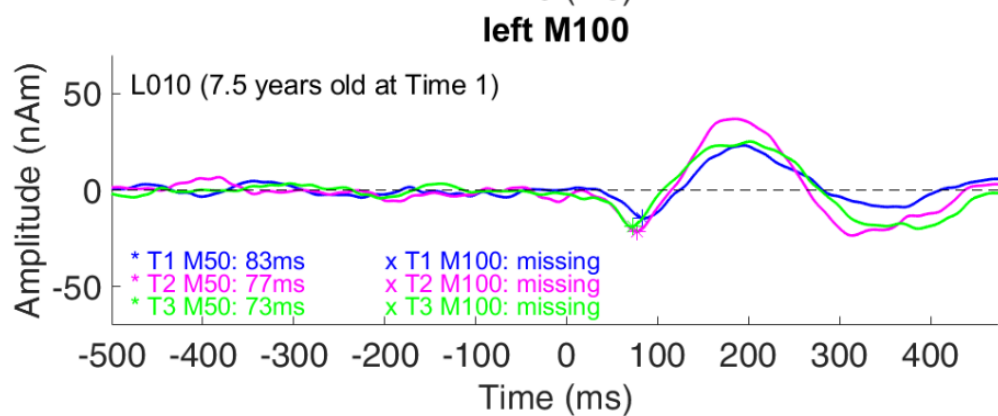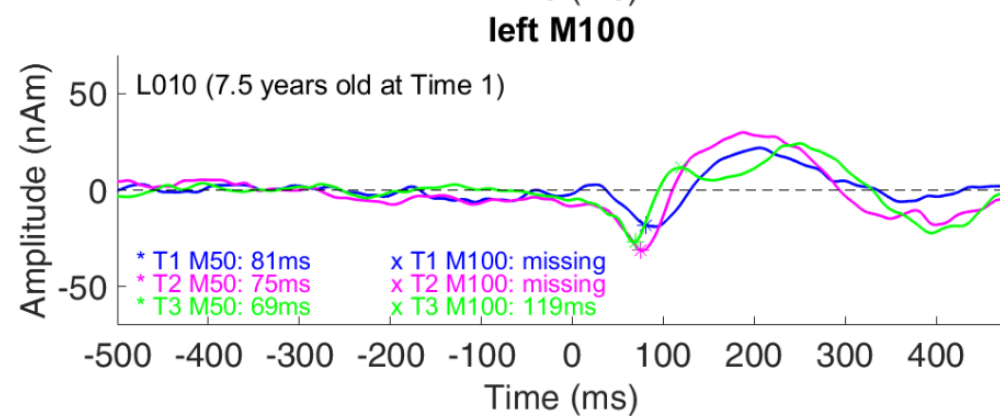

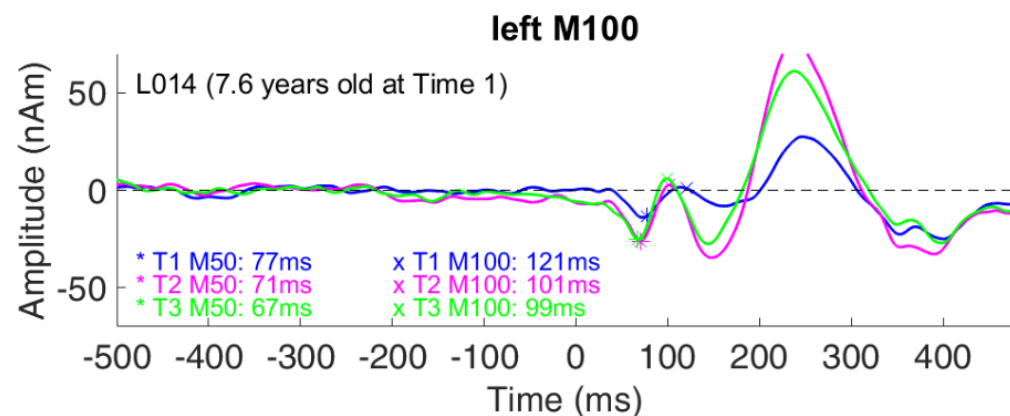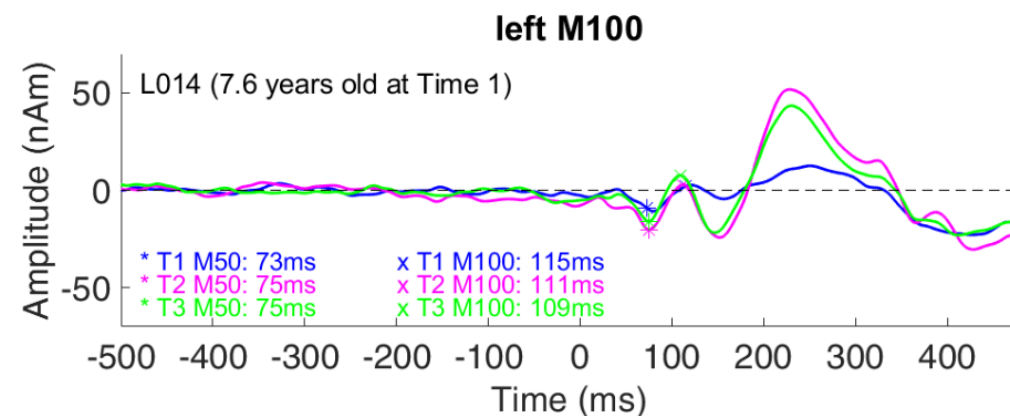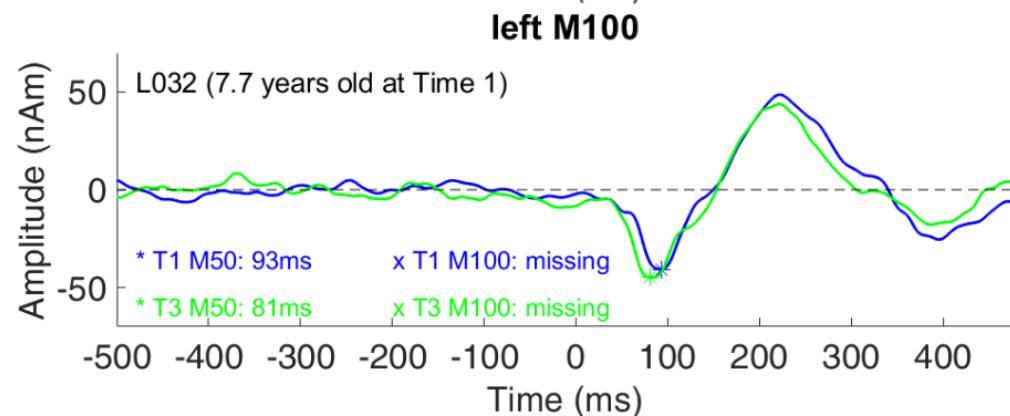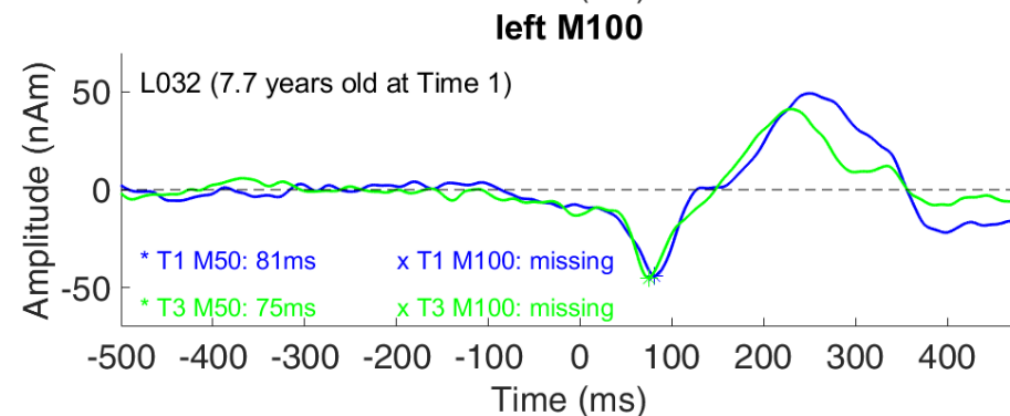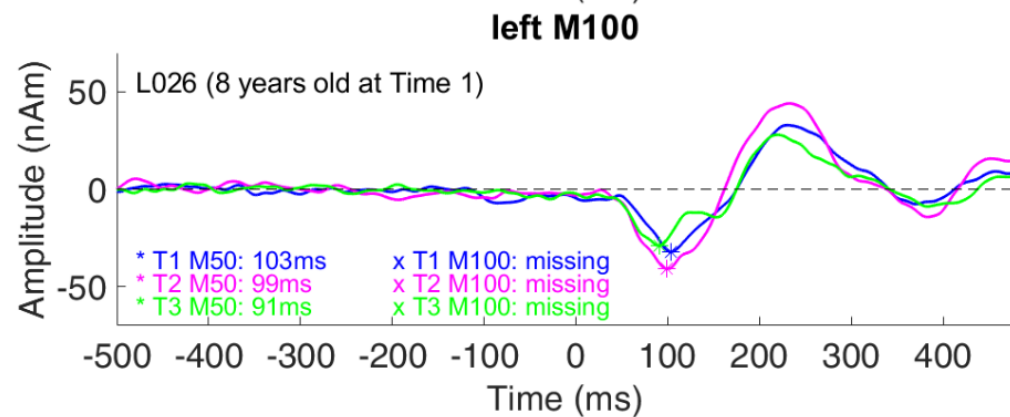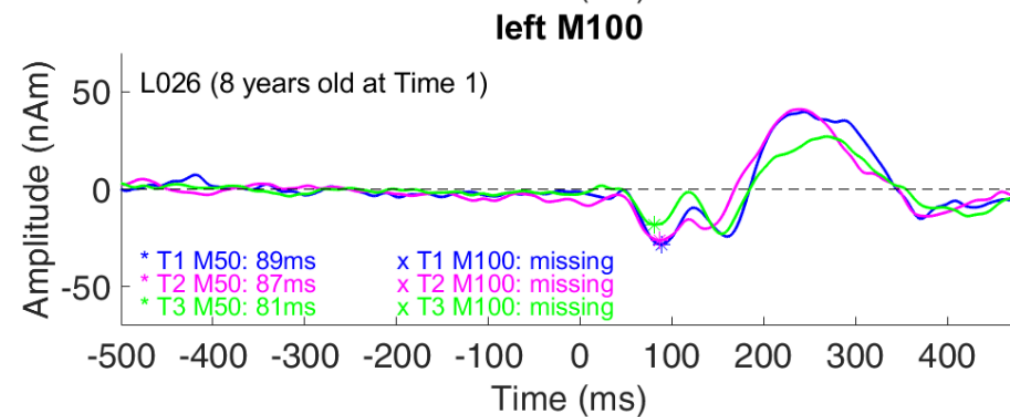

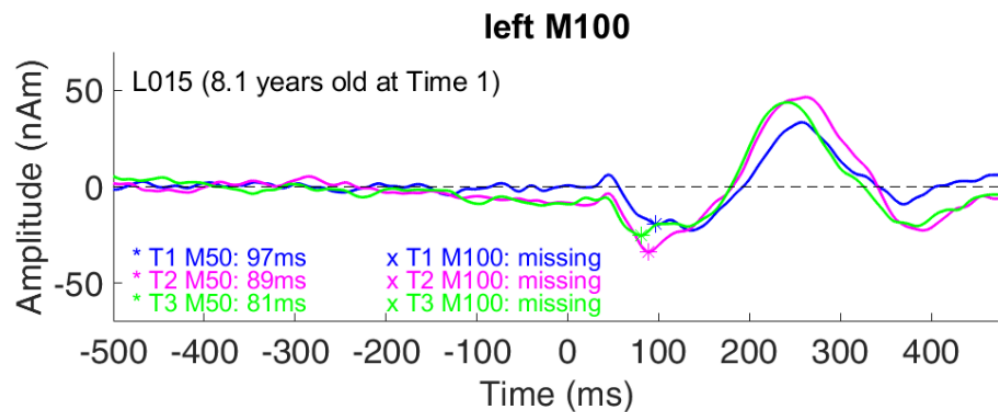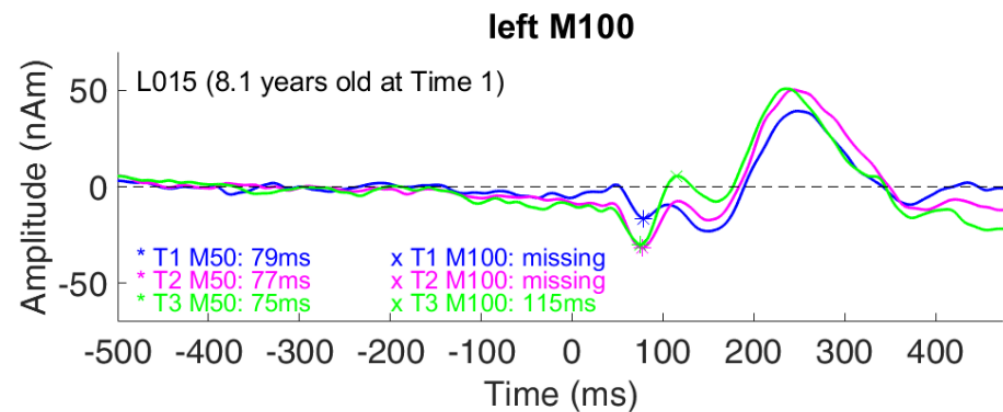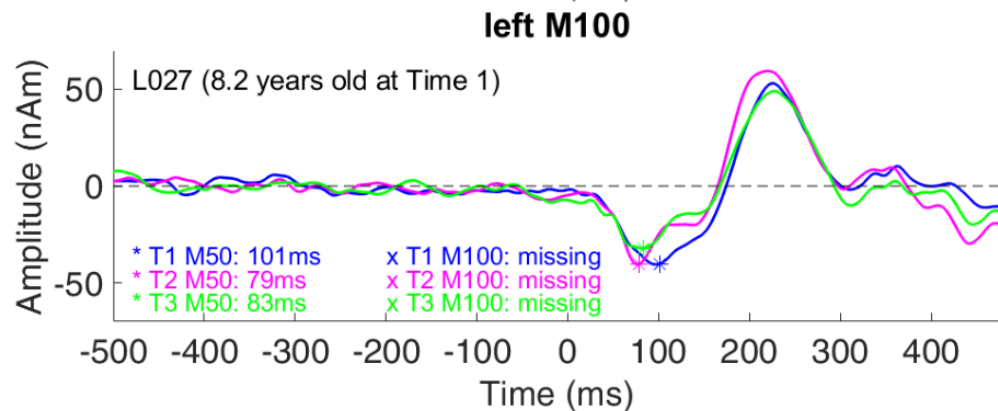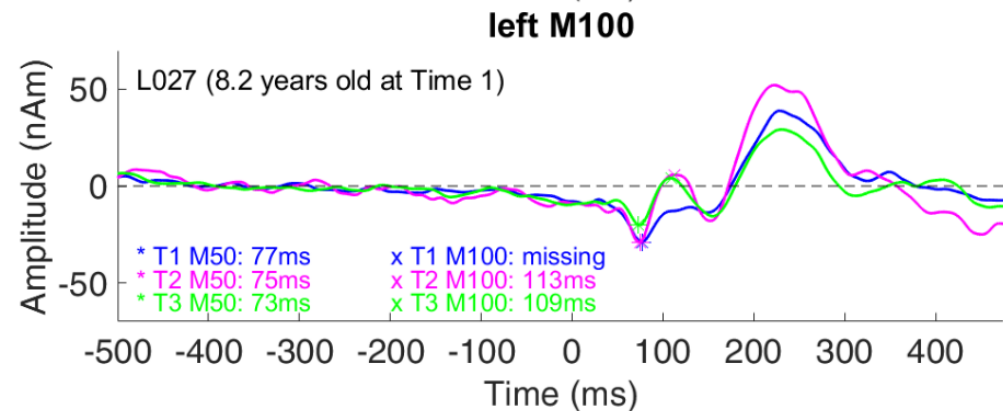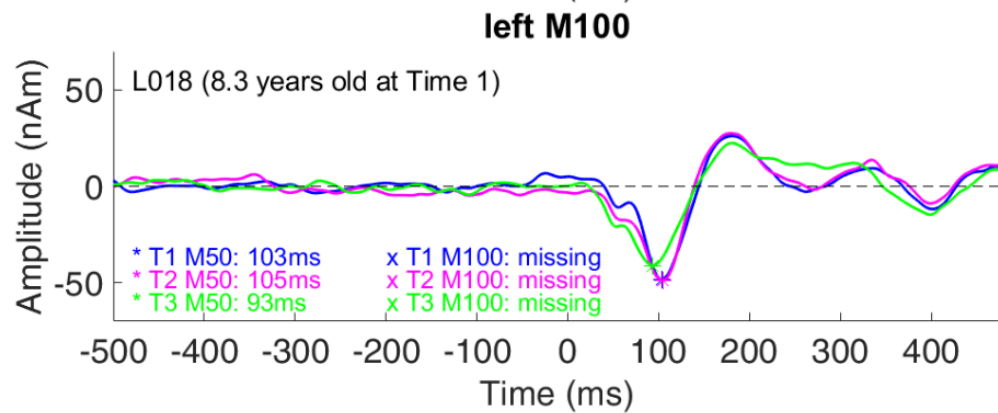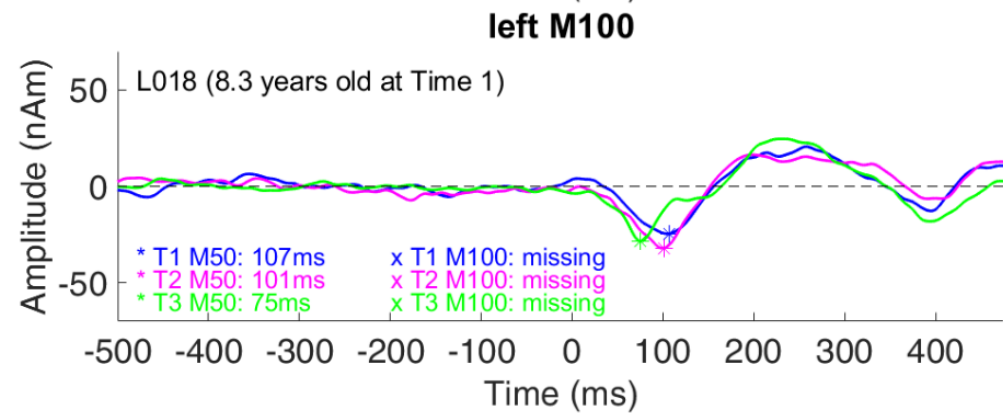

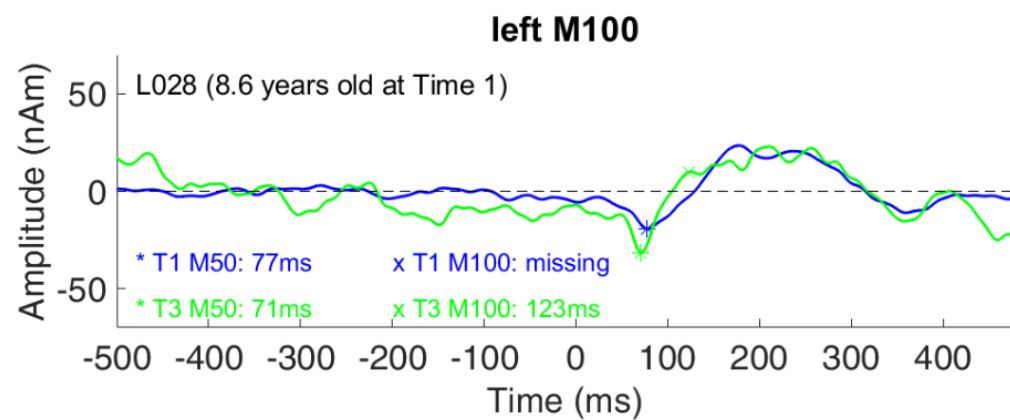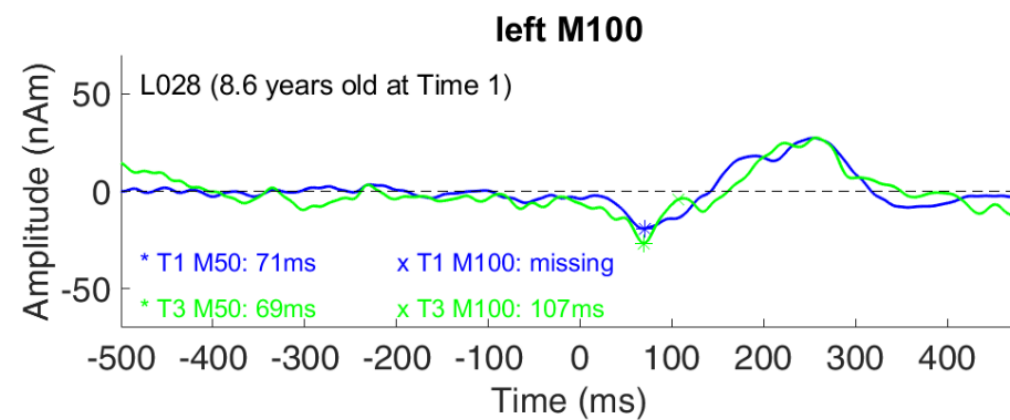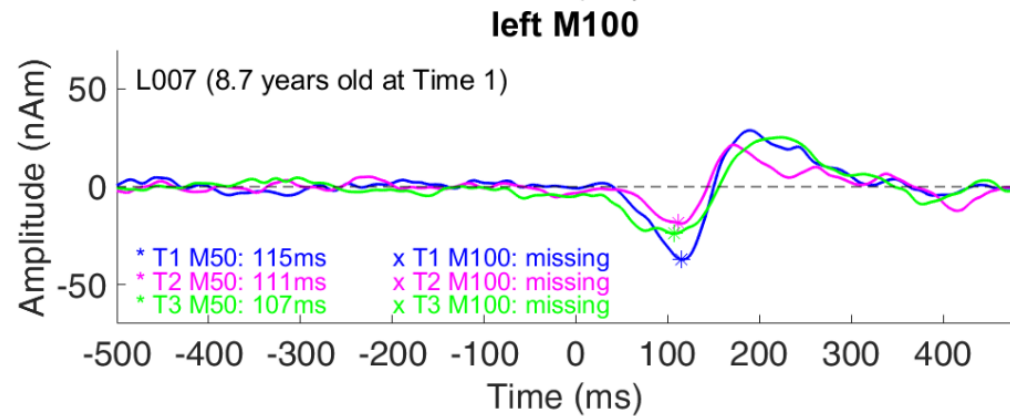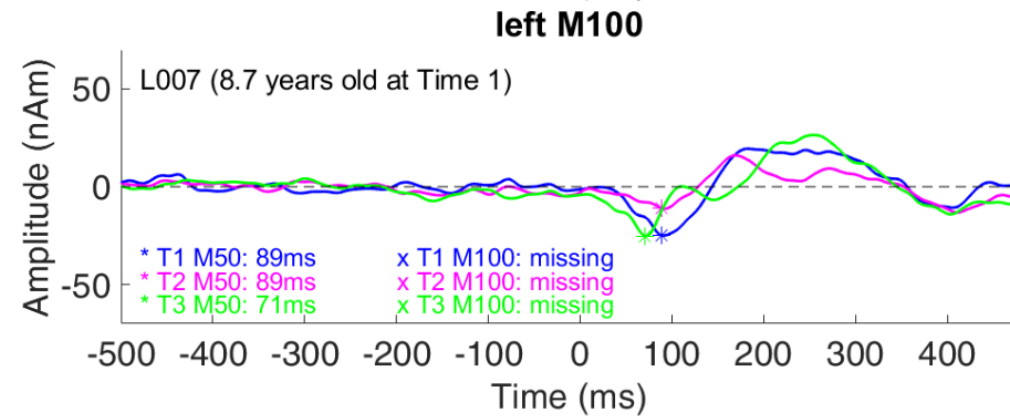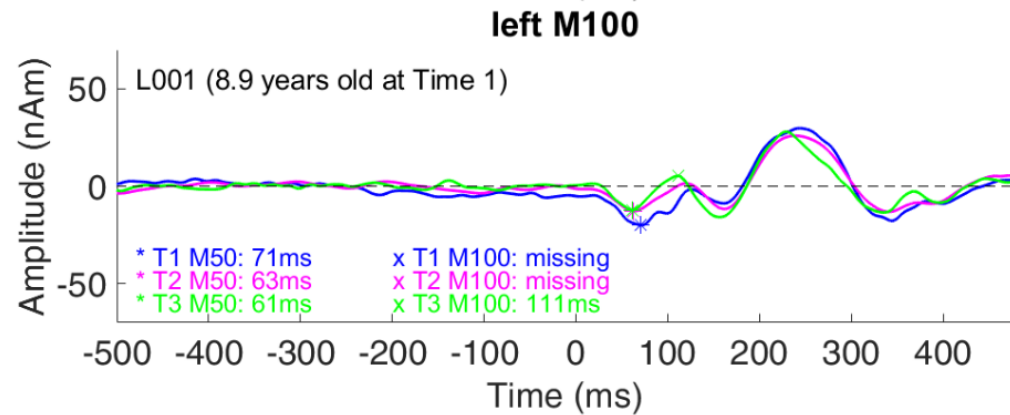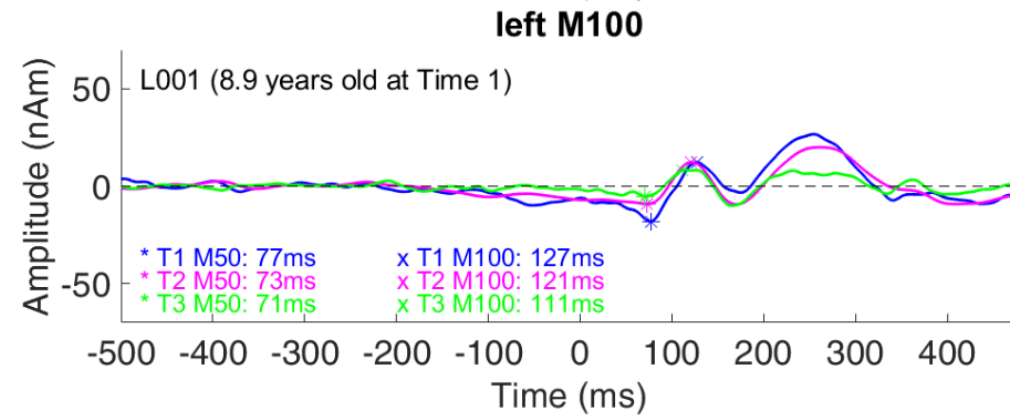

Supplement: Supplementary file 2 [file Data_Sheet_2.PDF]

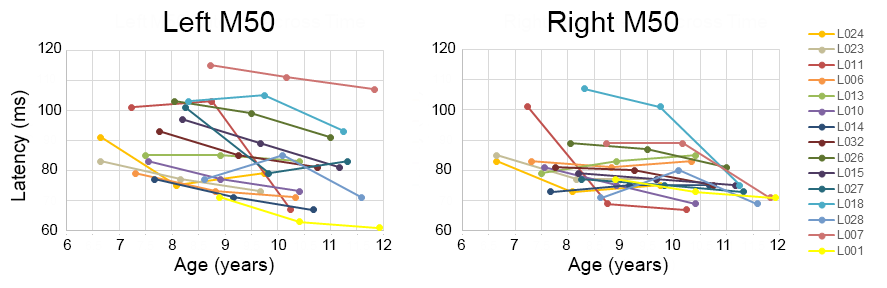

Supplement: Supplementary file 3 [file Image_1.TIF]
